# Controlled activation of cortical astrocytes modulates neuropathic pain-like behaviour

**DOI:** 10.1038/s41467-022-31773-8

**Published:** 2022-07-14

**Authors:** Ikuko Takeda, Kohei Yoshihara, Dennis L. Cheung, Tomoko Kobayashi, Masakazu Agetsuma, Makoto Tsuda, Kei Eto, Schuichi Koizumi, Hiroaki Wake, Andrew J. Moorhouse, Junichi Nabekura

**Affiliations:** 1 Division of Homeostatic Development, National Institute for Physiological Sciences, Okazaki, Japan; 2 Department of Anatomy and Molecular Cell Biology Graduate School of Medicine, Nagoya University, Nagoya, Japan; 3 Division of Multicellular Circuit Dynamics, National Institute for Physiological Sciences, Okazaki, Japan; 4 Department of Molecular and System Pharmacology, Graduate School of Pharmaceutical Sciences, Kyushu University, Fukuoka, Japan; 5 Division of Molecular Design, Research Center for Systems Immunology, Medical Institute of Bioregulation, Kyushu University, Fukuoka, Japan; 6 Department of Physiology, School of Allied Health Sciences, Kitasato University, Sagamihara, Kanagawa Japan; 7 Department of Neuropharmacology, Interdisciplinary Graduate School of Medicine, University of Yamanashi, Yamanashi, Japan; 8 GLIA Center, University of Yamanashi, Yamanashi, Japan; 9 Center of Optical Scattering Image Science Department of Systems Science, Kobe University, Kobe, Japan; 10 Department of Physiology, School of Medical Sciences, The University of New South Wales, Sydney, Australia; 11 Graduate School of Medicine, Nagoya University, Nagoya, Japan; 12 Department of Physiological Sciences, Graduate University for Advanced Studies, SOKENDAI, Hayama, Japan

**Keywords:** Synaptic plasticity, Astrocyte

## Abstract

Chronic pain is a major public health problem that currently lacks effective treatment options. Here, a method that can modulate chronic pain-like behaviour induced by nerve injury in mice is described. By combining a transient nerve block to inhibit noxious afferent input from injured peripheral nerves, with concurrent activation of astrocytes in the somatosensory cortex (S1) by either low intensity transcranial direct current stimulation (tDCS) or via the chemogenetic DREADD system, we could reverse allodynia-like behaviour previously established by partial sciatic nerve ligation (PSL). Such activation of astrocytes initiated spine plasticity to reduce those synapses formed shortly after PSL. This reversal from allodynia-like behaviour persisted well beyond the active treatment period. Thus, our study demonstrates a robust and potentially translational approach for modulating pain, that capitalizes on the interplay between noxious afferents, sensitized central neuronal circuits, and astrocyte-activation induced synaptic plasticity.

## Abstract (web summary)

Astrocytes may contribute to synaptic remodelling in the cortex in chronic pain states. Here the authors describe modulation of astrocyte activity to drive circuit reorganization in somatosensory cortex in mice, along with peripheral nerve block, which could be a potential therapeutic approach for the treatment of chronic pain.

## Introduction

Chronic pain remains as one of the leading causes of global disability, affecting around 20% of the adult population in the USA^1^. Novel approaches to reverse the mechanisms that cause the transition from acute to chronic pain are likely to have the greatest therapeutic impact^2^. Injury-induced maladaptive neural circuit plasticity, at both spinal and cortical levels, amplifies the noxious input and can render other sensory input as painful (allodynia)^3–5^. The somatosensory cortex (S1) encodes the location, intensity, and duration of nociceptive stimuli^6^, and may be rewired during the early post-injury period to contribute to chronic pain states^7,8^. Astrocytes are closely associated with neural circuit microstructure, and through both physical contacts and via secreted molecules can regulate a number of aspects of synaptic function, including synaptogenesis, spinogenesis, modulation of synaptic plasticity, and the elimination of spines and synapses^9,10^. Some of these astrocytic signalling pathways which are responsible for spinogenesis in development are reactivated following peripheral nerve injury to promote dendritic spine turnover, and thereby contribute to this remodelling of cortical pain circuits^11^. We proposed that corrective remodelling of these S1 circuit synapses may effectively reverse chronic pain. We achieved this by blocking the noxious peripheral afferent inputs while concurrently augmenting the activity of cortical astrocytes using transcranial direct current stimulation (tDCS) or the Designer Receptors Exclusively Activated by Designer Drugs (DREADD) system. This transient therapy induced rewiring of cortical circuit synapses and achieved a remission from allodynia-like behaviour that lasted well beyond the end of active treatment.

## Results

### tDCS-TTX therapy reverses allodynia-like behaviour in neuropathic model mice

tDCS has been widely applied to humans for a variety of neurological disorders, typically applied at current intensities between 1 and 4 mA over multiple sessions of 15–30 min duration^12^. Although tDCS is devoid of serious adverse effects, evidence for its efficacy in treating chronic pain is poor—at best providing only modest and temporary relief^12–14^. Conventional tDCS is believed to act via modulating spontaneous neuronal activity, but recent rodent studies demonstrated tDCS at lower current intensities can induce synchronized and widespread astrocytic Ca^2+^ transients in the absence of neuronal activation^15^. Using a similar protocol, we confirmed that a single tDCS session (0.1 mA for 10 min) applied over the cortex of awake mice (Supplementary Fig. 1) caused a significant and sustained increase in the frequency and amplitude of Ca^2+^ transients in S1 astrocytes (Supplementary Fig. 2a–c). This tDCS current intensity caused an approximate maximal level of astrocyte activation (Supplementary Fig. 3a). Larger current intensities were required to increase the integral of Ca^2+^ activity recorded from neuron somas (Supplementary Fig. 3b).

**Fig. 1:**
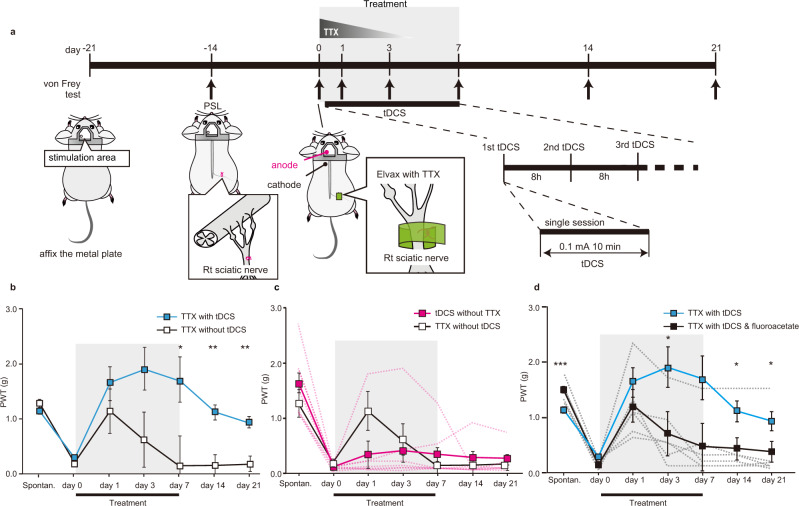
Astrocyte activation by transcranial direct current stimulation (tDCS) combined with transient nerve block reverses mechanical allodynia-like behaviour. **a** Schematic of the experimental schedule. The start of tDCS-TTX (or control) treatment was designated as “day 0”. Surgery for head-plate fixation and PSL at the right hind limb were performed at day-21 and day-14, respectively. Mechanical paw withdrawal thresholds (von Frey) were measured (arrows) before injury (day-14), and before (day 0), during (day 0–day 7), and after (day 8 onwards) treatment. tDCS (black bar) for activating astrocytes (constant 0.1 mA current for 10 min, see Supplementary Fig. 1 for more detail) was administered at 8-hourly intervals from day 0 to day 7 (21 sessions in total). TTX (graded triangle) was continuously administered to the injured right sciatic nerve via elution from an Elvax cuff preparation from day 0 to day 5. **b**–**d** Graphs plotting mechanical paw withdrawal thresholds as mean ± SEM at various time-points before PSL (Spontan.), and before (day 0), during (day 1–day 7) and after (day 8 onwards) the TTX with tDCS treatment period (grey shading) or various other control treatments. Dashed-lines represent individual mice. For all graphs separately, statistical significance was tested using a two-way repeated measures ANOVA, followed by Bonferroni post-hoc tests comparing treatment cohorts at each time-point, *p < 0.05, **p < 0.01, ***p < 0.001. Source data are provided as a Source Data file. **b** Combined TTX with tDCS treatment (blue, *n* = 7 mice), but not TTX treatment alone (white, *n* = 4 mice), reversed mechanical allodynia-like behaviour, with mechanical paw withdrawal thresholds returning to pre-PSL levels (see Supplementary Fig. 4a for values from individual mice). **c** Separate administration of tDCS (magenta, *n* = 8 mice) failed to reverse mechanical allodynia-like behaviour. Note the TTX without tDCS data (white, *n* = 4 mice) in this panel replots the same data as shown in (**b**) for comparison. **d** tDCS with TTX & fluoroacetate (black, *n* = 6 mice) failed to reverse mechanical allodynia-like behaviour. Elvax with fluoroacetate was applied onto S1 on day 0. Note the TTX with tDCS data (blue, *n* = 7 mice) in this panel replots the same data as shown in (**b**) for comparison.

We asked if modulating astrocyte activity could impact chronic pain behaviour and used partial sciatic nerve ligation (PSL)^16^ to induce a sustained allodynia-like behaviour. Two weeks after PSL, we used von Frey hairs to confirm the presence of mechanical allodynia-like behaviour and assigned this as “day 0” in our protocol (Fig. 1a). Single sessions of tDCS were then applied over the subsequent week at regular 8-hourly intervals. We further reasoned that blocking noxious afferent activity from the injured nerve may also be required to reverse chronic allodynia-like behaviour, so simultaneously delivered tetrodotoxin (TTX) locally to the sciatic nerve via an implanted Elvax drug elution cuff (Fig. 1a). Prior to any treatment (day 0 in Fig. 1b–d), all mice displayed mechanical allodynia-like behaviour following PSL as shown by decreases in paw withdrawal thresholds. In PSL mice receiving TTX application alone, allodynia-like behaviour was transiently ameliorated (day 1 in Fig. 1b, c) with paw withdrawal thresholds gradually decreasing again to day 0 (post PSL) levels as TTX elution subsided^11^, indicating return of mechanical allodynia-like behaviour (Fig. 1b, c, TTX without tDCS (white, *n* = 4), mean ± SEM of paw withdrawal thresholds (g); Spontan.: 1.3 ± 0.16, day 0: 0.21 ± 7.0E−2, day 1: 1.1 ± 0.44, day 3: 0.63 ± 0.19, day 7: 0.17 ± 3.2E−2, day 14: 0.17 ± 3.2E−2, day 21: 0.20 ± 2.6E−2). However, in PSL mice receiving combined TTX with tDCS, paw withdrawal thresholds were sustained at pre-injury levels, even two weeks after stoppingtherapy (Fig. 1b, d, TTX with tDCS, (blue, *n* = 7), mean ± SEM of paw withdrawal thresholds (g), Spontan.: 1.1 ± 2.4E−2, day 0: 0.28 ± 4.6E−2, day 1: 1.6 ± 0.29, day 3: 1.9 ± 0.45, day 7: 1.7 ± 0.50, day 14: 1.1 ± 0.17, day 21: 0.94 ± 0.12). The post-treatment paw-withdrawal thresholds were significantly different between the two cohorts (TTX with tDCS vs TTX without tDCS, a two-way repeated measures ANOVA, the interaction effect: F(1.8, 1.6E+1) = 3.6, *p* = 5.5E−2, the main effect: F(1, 9) = 6.1, *p* = 3.5E−2, the main effect: *p* = 4.8E−2; followed by Bonferroni post hoc tests, Spontan. (*p* = 2.7E−1), day 0 (*p* = 3.9E−1), day 1(*p* = 3.5E−1), day 3 (*p* = 7.2E−2), day 7 (*p* = 4.7E−2), day 14 (*p* = 2.4E−3), day 21(*p* = 1.4E−3)) indicating reversal of allodynia-like behaviour by combined TTX with tDCS treatment. Individual mice paw withdrawal thresholds for the data in Fig. 1b are shown in Supplementary Fig. 4a. Thermal allodynia-like behaviour was also reversed by combined tDCS with TTX treatment, without any effects on withdrawal thresholds in the uninjured contralateral paw (Supplementary Fig. 5). Conventional therapeutic tDCS, i.e., without simultaneous blockade of noxious inputs with TTX, was ineffective in providing lasting pain relief (Fig. 1c, tDCS without TTX (magenta, *n* = 8), mean ± SEM of paw withdrawal thresholds (g), Spontaneous paw withdrawal thresholds (g): 1.6 ± 0.2, day 0: 0.13 ± 1.8E−2, day 1: 0.36 ± 0.21, day 3: 0.42 ± 0.22, day 7: 0.36 ± 0.15, day 14: 0.30 ± 0.14, day 21: 0.26 ± 0.11; TTX without tDCS (same as Fig. 1b) vs tDCS without TTX, a two-way repeated-measures ANOVA: the interaction effect, F(1.7, 1.7E+1) = 3.2, *p* = 0.74, the main effect F(1,10) = 6.5E−1, *p* = 0.84). To confirm the locus of the therapeutic target in tDCS, we blocked S1 astrocyte activation using the metabolic inhibitor, fluoroacetate, applied via an Elvax cuff placed on the surface of S1^17^. When local S1 astrocytes were inhibited in this way, TTX with tDCS failed to reverse allodynia-like behaviour (Fig. 1d, TTX with tDCS & fluoroacetate (black, *n* = 6), mean ± SEM of paw withdrawal thresholds (g), spontan.: 1.5 ± 7.4E−2, day 0: 0.17 ± 3.2E−2, day 1: 1.2 ± 0.25, day 3: 0.72 ± 0.24, day 7: 0.48 ± 0.22, day 14: 0.45 ± 0.22, day 21: 0.39 ± 0.23, TTX with tDCS (same as Fig. 1b) vs TTX with tDCS & fluoroacetate, a two-way repeated measures ANOVA: the interaction effect F(1.7, 1.9E+1) = 4.4, *p* = 3.0E−2; followed by Bonferroni post-hoc tests, Spontan. (*p* = 3.6E−4), day 0 (*p* = 7.0E−2), day 1 (*p* = 3.1E−1), day 3 (*p* = 4.8E−2), day 7 (*p* = 5.6E−2), day 14 (*p* = 3.2E−2), day 21(*p* = 4.8E−2)). Together our results show that a brief treatment regime combining tDCS (configured to induce S1 astrocyte activation) with TTX-blockade of noxious afferents, i.e., TTX with tDCS therapy, is able to mediate a lasting adaptive response to reverse mechanical allodynia.

### Specific chemogenic activation of S1 astrocytes also reverses allodynia-like behaviour

**Fig. 2:**
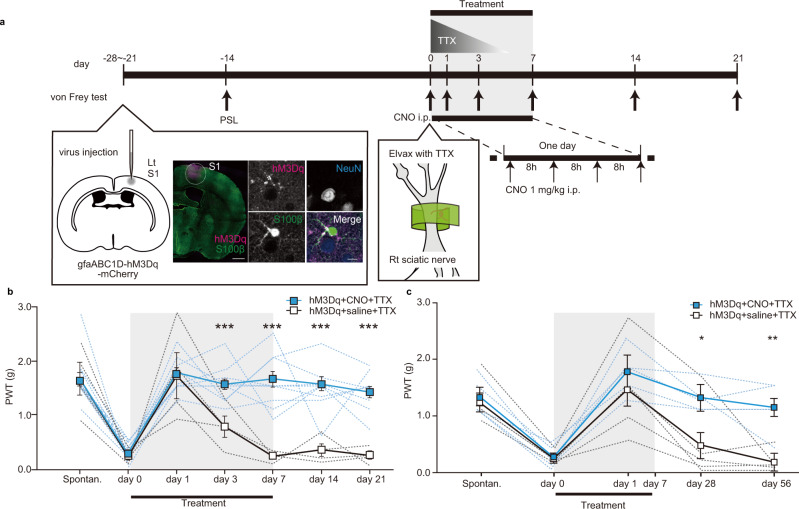
Chemogenetic activation of targeting S1 astrocytes combined with transient nerve block reverses mechanical allodynia-like behaviour. **a** Schematic of the experimental schedule. The start of CNO-TTX (or control) treatment was designated as “day 0”. Injection of gfaABC1D-hM3Dq-mCherry AAV into the PSL-contralateral S1 was performed between day −28 to day −21. Immunohistochemistry confirmed widespread expression (see Supplementary Fig. 6 for quantification) of hM3Dq (mCherry tag) in S1 astrocytes (S100β staining) with no off-target expression in S1 neurons (NeuN staining). 1 mg/kg CNO (black bar) was injected at 8-hourly intervals from day 0 to day 7 (21 injections in total). PSL surgery, measurement of mechanical paw withdrawal thresholds (arrows), and TTX administration (graded triangle) were performed as in Fig. 1. Left figure scale bar = 500 μm. Right figure scale bar = 10 μm. **b**, **c** Graphs plotting mechanical paw withdrawal thresholds as mean ± SEM at various time-points before PSL (Spontan.) and before (day 0), during (day 1–day 7), and after (day 8 onwards) the TTX with CNO treatment period (grey shading) or control TTX with saline treatment. For all graphs separately, statistical significance was tested using a two-way repeated measures ANOVA, followed by Bonferroni post hoc tests comparing treatment cohorts at each time-point, *p < 0.05, **p < 0.01, ***p < 0.001. Source data are provided as a Source Data file. **b** Combined TTX with CNO (1.0 mg/kg) treatment (blue, *n* = 13 mice), but not control TTX with saline treatment (white, *n* = 4 mice) reversed mechanical allodynia-like behaviour. **c** The therapeutic amelioration of mechanical allodynia-like behaviour by combined TTX with CNO (1.0 mg/kg) treatment (blue, *n* = 5 mice), but not control TTX with saline treatment (white, *n* = 5 mice), persisted well beyond the end of the treatment period.

tDCS is known to broadly activate astrocytes across the cortex both ipsilateral and contralateral to the stimulating anode^15^. To more specifically determine the locus of the therapeutic effect, we used the DREADD system^18^ as a different and more targeted approach (Fig. 2a). We expressed the hM3Dq receptor in S1 astrocytes by localized AAV injection (Fig. 2a and Supplementary Fig. 6), achieving expression in ~89% of L1-3 astrocytes in S1 (based on a sample of 392 astrocytes from 5 mice). A single intraperitoneal injection of clozapine N-oxide (CNO; 1.0 mg/kg, i.p.), which activates this hM3Dq receptor, also increased astrocytic Ca^2+^ transients. Again this astrocyte activation was seen without any concurrent increase in Ca^2+^ transients in S1 neuron somas (Supplementary Fig. 7). The increase in the Ca^2+^ transient response was specific to S1 astrocytes; the activity of astrocytes in M1 was not affected by CNO (Supplementary Fig. 8). Repeated CNO administration (over 4 days, every 8 h) induced up-regulation of glial fibrillary acidic protein (GFAP) in S1 astrocytes (Supplementary Fig. 9), indicating they adopted an activated phenotype. Consistent with the rapid distribution of CNO^19^, astrocytic Ca^2+^ activity peaked immediately after administration but then showed a sustained increase from 3 h onwards (Supplementary Fig. 10), perhaps reflecting prolonged signalling or maximal CNO accumulation in brain tissues^19^. Given that these sustained increases in astrocytic Ca^2+^ were comparable to those seen with tDCS, we used this CNO dose in mice with established PSL-induced allodynia-like behaviour. We administered CNO at 8-hourly intervals for 1 week, whilst again simultaneously blocking noxious afferent input from the injured sciatic nerve with local TTX elution from the Elvax cuff (Fig. 2a). As before, TTX administration immediately restored paw withdrawal thresholds to pre-injury levels (Fig. 2b), but this was only transient in mice treated with concurrent saline injections (hM3Dq + saline + TTX (white, *n* = 4), mean ± SEM of paw withdrawal thresholds (g), Spontan.: 1.7 ± 0.30, day 0: 0.24 ± 6.8E−2, day 1: 1.7 ± 0.42, day 3: 0.78 ± 0.19, day 7: 0.25 ± 4.3E−2, day 14: 0.36 ± 0.12, day 21: 0.26 ± 7.4E−2). In contrast, mice given TTX and concurrent CNO injections continued to maintain pre-injury withdrawal thresholds (hM3Dq + CNO + TTX (blue, *n* = 13), mean ± SEM of paw withdrawal thresholds (g), Spontan.: 1.7 ± 0.11, day 0: 0.32 ± 4.6E−2, day 1: 1.8 ± 9.3E−2, day 3: 1.6 ± 9.0E−2, day 7: 1.7 ± 0.15, day 14: 1.6 ± 0.14, day 21: 1.5 ± 0.10). The post-treatment paw withdrawal thresholds were significantly different between the two cohorts (hM3Dq + CNO + TTX vs hM3Dq + saline + TTX, a two-way repeated measures ANOVA: interaction effect, F(3.7, 5.1E+1) = 7.7, *p* = 9.5E−5; followed by Bonferroni post hoc tests, Spontan. (*p* = 9.7E−1), day 0 (*p* = 4.9E−1), day 1 (*p* = 8.5E−1), day 3 (*p* = 1.4E−3), day 7 (*p* = 7.7E−5), day 14 (*p* = 1.4E−4), and day 21 (*p* = 2.3E−5)) indicating a sustained reversal of allodynia-like behaviour mediated by TTX with CNO treatment. This relief from allodynia-like behaviour could last for at least 2 months, the latest time-point that we checked (Fig. 2c, mean ± SEM of paw withdrawal thresholds (g), hM3Dq + CNO + TTX (blue, *n* = 5), Spontan.: 1.3 ± 0.15, day 0: 0.28 ± 9.1E−2, day 1: 1.8 ± 0.18, day 28: 1.3 ± 0.12, day 56: 1.2 ± 0.20; hM3Dq + saline + TTX, (white, *n* = 5), Spontan.: 1.2 ± 0.17, day 0: 0.25 ± 4.7E−2, day 1: 1.5 ± 0.37, day 28: 0.48 ± 0.31, day 56: 0.19 ± 9.0E−2; hM3Dq + CNO + TTX vs hM3Dq + saline + TTX, a two-way repeated measures ANOVA: main effect, interaction effect, F(1.6, 13) = 3.8, *p* = 0.58, main effect, F(1.6, 13) = 23, *p* = 8.9E−5; followed by Bonferroni post-hoc tests, Spontan (*p* = 6.6E−1), day0(*p* = 8.1E−1), day 1 (*p* = 4.7E−1), day 28 (*p* = 3.4E−2), day 56 (*p* = 2.0E−3)). Importantly, both CNO treatment by itself (with astrocytic mCherry expression as a control for hM3Dq), and CNO in mice expressing hM3Dq in astrocytes but not given concurrent TTX, failed to reverse allodynia-like behaviour (Supplementary Fig. 11a; mean ± SEM of withdrawal thresholds(g), mCherry + CNO + TTX (*n* = 5), Spontan.: 1.7 ± 0.21, day 0: 0.31 ± 6.4E−2, day 1: 1.5 ± 0.20, day 3: 0.94 ± 0.18, day 7: 0.61 ± 0.18, day 14: 0.67 ± 0.22, day 21: 0.20 ± 0.20; hM3Dq + CNO + vehicle, (*n* = 8), Spontan.: 1.6 ± 0.17, day 0: 0.15 ± 5.0E−2, day 1: 0.21 ± 0.16, day 3: 0.33 ± 0.14, day 7: 0.11 ± 0.15, day 14: 0.31 ± 0.18, day 21: 0.23 ± 0.15). Furthermore, lower doses of CNO that caused significantly smaller astrocyte activation, did not reverse the allodynia-like behaviour following PSL (Supplementary Fig. 12). We also examined whether the treatment protocol, using behaviour assays to compare, hM3Dq + CNO + TTX and hM3Dq + saline + TTX mice cohorts (Supplementary Fig. 13). In an open field assay, there were no significant differences between cohorts in the total distance travelled, the speed of travel, or the time spent in the centre zone of the open field, suggesting that the treatment protocol did not affect anxiety and locomotion behaviours. In a rotarod assay, there was no difference in the time to fall from the rotarod, confirming that the reversal of allodynia-like behaviour was not due to altered motor coordination. In conclusion, a transient post-injury regime of CNO-TTX therapy or tDCS-TTX therapy are both able to prevent long-term allodynia-like behaviour, indicating that the key locus of the therapeutic effect is the selective activation of S1 astrocytes concomitant with blockade of noxious afferent input.

### The therapy preferentially eliminates dendritic spines associated with noxious circuits

**Fig. 3:**
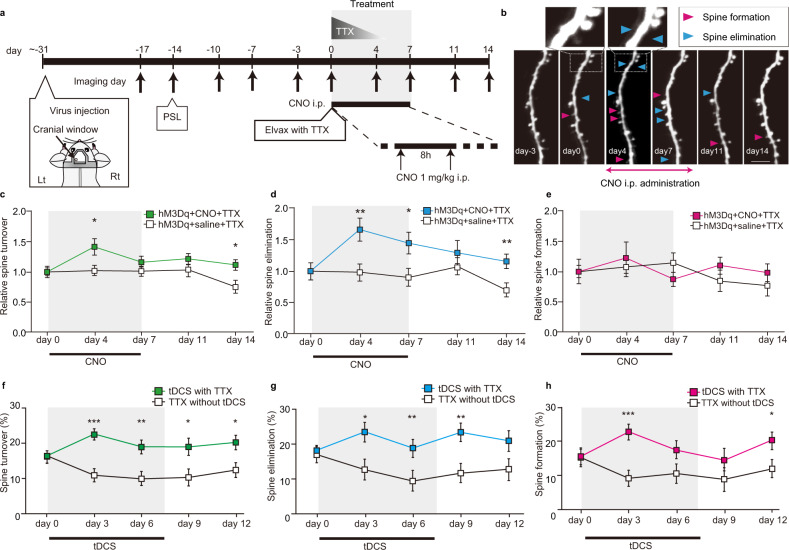
Increased dendritic spine elimination accompanies the reversal of mechanical allodynia-like behaviour by CNO-TTX or tDCS-TTX therapy. **a** Schematic of the experimental schedule. The start of CNO-TTX (or control) treatment was designated as “day 0”. In vivo 2-photon imaging of spine dynamics was performed (arrows) before injury (day 17–day 14), and before (day 10–day 0), during (day 1–day 7) and after (day 8 onwards) CNO-TTX treatment. Surgery for head-plate fixation, gfaABC1D-hM3Dq-mCherry AAV injection and PSL, and measurement of mechanical paw withdrawal thresholds (arrows), and TTX administration (graded triangle) were performed as in Fig. 2. **b** Repeated imaging of the same dendritic segment from a layer 5 pyramidal neuron in the left (PSL-contralateral) S1 before (day 3–day 0), during (day 4–day 7) and after (day 11–day 14) CNO-TTX treatment. Arrowheads indicate spine formation (magenta) and spine elimination (blue). Scale bar = 5 μm. Insets show magnified regions as indicated by dashed boxes. The experiments showed successful reproducibility. **c**–**h** Graphs plotting measures of spine dynamics as mean ± SEM at various time-points before (day 0), during (day 1–day 7) and after (day 8 onwards) the treatment period (grey shading). For all graphs separately, statistical significance was tested using a two-way repeated measures ANOVA, followed by Bonferroni post hoc tests comparing treatment cohorts at each time-point, *p < 0.05, **p < 0.01, ***p < 0.001. Source data are provided as a Source Data file. **c**–**e** The treatment period refers to CNO-TTX (*n* = 19 dendrites from 5 mice) or control saline-TTX (*n* = 16 dendrites from 7 mice) therapy administered as in Fig. 2. Spine dynamics are reported as values normalized to day 0 (see Supplementary Fig. 14 for absolute values and explanatory note). **c** Spine turnover, CNO-TTX (green), control saline-TTX (white). **d** Spine elimination, CNO-TTX (blue), control saline-TTX (white). **e** Spine formation, CNO-TTX (magenta), control saline-TTX (white). **f**–**h** The treatment period refers to tDCS-TTX (*n* = 16 dendrites from 3 mice) or control TTX-alone (*n* = 14 dendrites from 5 mice) therapy administered as in Fig. 1. Spine dynamics are reported as absolute values. **f** Spine turnover, tDCS-TTX (green), control TTX-alone (white). **g** Spine elimination, tDCS-TTX (blue), control TTX-alone (white). **h** Spine formation, tDCS-TTX (magenta), control TTX-alone (white).

**Fig. 4:**
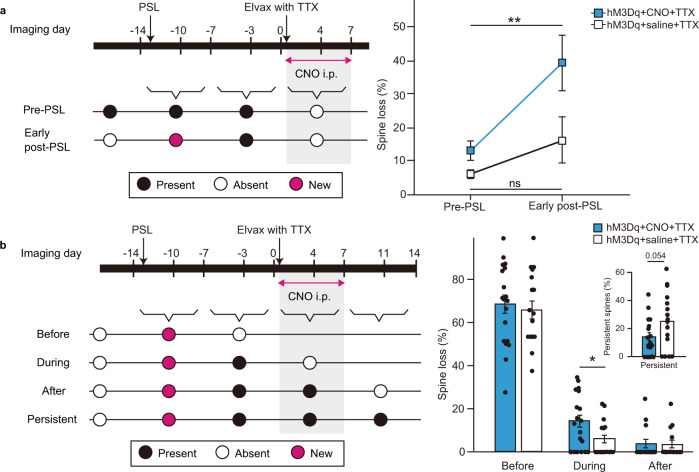
CNO-TTX therapy enhances the preferential elimination of dendritic spines that are likely associated with maladaptive noxious circuits. **a** Left panel: Schematic categorizing spines eliminated during CNO-TTX (or control) treatment. “Pre-PSL” spines were present before PSL and before starting CNO-TTX treatment. “Early post-PSL” spines appeared in the week after PSL and were still present before starting CNO-TTX treatment. Right panel: Graph plotting the proportion of eliminated pre-PSL or early post-PSL spines during CNO-TTX (blue, 19 dendrites from 5 mice) or control saline-TTX (white, 16 dendrites from 7 mice) treatment as mean ± SEM. Statistical significance was tested using a two-way ANOVA, followed by Bonferroni post hoc tests comparing spine categories within each treatment cohort, **p < 0.01. Source data are provided as a Source Data file. **b** Left panel: Schematic further categorizing early post-PSL spines based on whether they were eliminated “Before”, “During” or “After” CNO-TTX (or control) treatment or were “Persistent” (not eliminated). All of these spine categories are likely associated with maladaptive noxious circuits. Right panel: Graphs plotting the proportional representation of each category of early post-PSL spines for CNO-TTX (*n* = 19 dendrites from 5 mice with 171 spines in total) and control saline-TTX (*n* = 16 dendrites from 7 mice with 119 spines in total), as mean ± SEM with single points representing individual dendrites. Statistical significance was tested using an unpaired two-sided *t* test comparing treatment cohorts for each spine category, *p < 0.05. Source data are provided as a Source Data file.

We next examined the mechanisms by which the combination therapy was able to induce this seemingly permanent reversal of the chronic pain-like response. Previous studies have demonstrated that transient and discrete astrocyte activation in the first week following PSL triggers a period of increased spine formation and elimination in S1^11,20^, which may represent maladaptive plasticity of cortical neural circuits that engenders nociceptive responses to previously non-noxious sensory stimuli. We therefore hypothesized that subsequently augmenting astrocyte activity, while simultaneously blocking noxious afferent input, could re-awaken spine plasticity and induce an adaptive rewiring of this maladapted circuitry. We therefore imaged S1 layer 5 pyramidal neuron dendritic spines, starting prior to PSL and again during and after astrocyte activation (Fig. 3a, b, see also Supplementary Fig. 14 for extended data). Activation of astrocytes by CNO, combined with peripheral TTX treatment, induced an increase in relative spine turnover during treatment, which was associated with an increase in relative spine elimination (Fig. 3a–e, hM3Dq + CNO + TTX, (*n* = 19 dendrites from 5 mice), mean ± SEM of spine dynamics measure; spine turnover (green, Fig. 3c), day 0: 1.0 ± 9.3E−2, day 4: 1.4 ± 0.12, day 7: 1.1 ± 8.9E−2, day 11: 1.2 ± 9.8E−2, day 14: 1.1 ± 0.11; spine elimination (blue, Fig. 3d), day 0: 1.0 ± 0.11, day 4: 1.7 ± 0.15, day 7: 1.5 ± 0.16, day 11: 1.4 ± 0.17, day 14: 1.2 ± 0.13; spine formation (magenta, Fig. 3e), day 0: 1.0 ± 0.15, day 4: 1.2 ± 0.22, day 7: 0.88 ± 0.13, day 11: 1.1 ± 0.15, day 14: 1.0 ± 0.15). In contrast, mice with peripheral TTX application alone, had spine dynamics that appeared more stable (Fig. 3c–e, hM3Dq + saline + TTX (*n* = 16 dendrites from 7 mice), mean ± SEM of spine dynamics measure; spine turnover (white, 3c), day 0: 1.0 ± 0.10, day 4: 1.0 ± 0.13, day 7: 1.0 ± 9.7E−2, day 11: 0.97 ± 0.11, day 14: 0.72 ± 0.12); spine elimination (white, 3d), day 0: 1.0 ± 0.12, day 4: 0.96 ± 0.16, day 7: 0.87 ± 0.18, day 11: 1.1 ± 0.19, day 14: 0.67 ± 0.17; spine formation (white, 3e), day 0: 1.0 ± 0.16, day 4: 1.1 ± 0.24, day 7: 1.2 ± 0.14, day 11: 0.85 ± 0.16, day 14: 0.78 ± 0.17). Similarly, combined TTX with tDCS increased spine turnover during treatment which was associated with increased elimination and formation (Fig. 3f–h, TTX with tDCS (*n* = 16 dendrites from 3 mice), mean ± SEM of spine dynamics measure; spine turnover (green, Fig. 3f), day 0: 16 ± 1.7%, day 3: 22 ± 1.7%, day 6: 19 ± 1.9%, day 9: 19 ± 2.3%, day 12: 20 ± 2.0%; spine elimination (blue, Fig. 3g), day 0: 18 ± 1.9%, day 3: 23 ± 2.6%, day 6: 20 ± 2.5%, day 9: 23 ± 2.5%, day 12: 21 ± 2.8%; spine formation (magenta, Fig. 3h), day 0: 15 ± 2.3%, day 3: 22 ± 2.2%, day 6: 18 ± 2.6%, day 9: 15 ± 3.2%, day 12: 20 ± 2.4%). Again spine dynamics in TTX without tDCS (control) mice appeared more stable, possibly slower (Fig. 3f–h, TTX without tDCS, (*n* = 14 dendrites from 5 mice), spine turnover (white, 3 f), day 0: 16 ± 1.8%, day 3: 11 ± 1.9%, day 6: 9.9 ± 2.0%, day 9: 10 ± 2.5%, day 12: 12 ± 2.2%; spine elimination (white, 3 g), day 0: 17 ± 1.9%, day 3: 13 ± 2.8%, day 6: 9.4 ± 2.6%, day 9: 12 ± 2.7%, day 12: 13 ± 3.0%; spine formation (white, 3 h), day 0: 15 ± 2.5%, day 3: 9.1 ± 2.4%, day 6: 11 ± 2.4%, day9: 8.9 ± 3.4%, day 12: 12 ± 2.5%). Notably, spine turnover and elimination (but not formation) rates were significantly increased in mice receiving CNO-TTX therapy as compared to mice receiving saline-TTX therapy(Fig. 3c, d, hM3Dq + CNO + TTX vs hM3Dq + saline + TTX, a two-way repeated ANOVA followed by Bonferroni post hoc tests where appropriate; spine turnover, the interaction effect: F(4, 1.3E+2) = 1.5, *p* = 2.2E−1, the main effect for group: F(1, 33) = 6.4, *p* = 1.6E−2; followed by Bonferroni post hoc test, day 0 (*p* = 1.0), day 4 (*p* = 3.1E−2), day 7 (*p* = 3.9E−1), day 11 (*p* = 1.1E−1), and day 14 (*p* = 3.2E−2); spine elimination, the interaction effect: F(4, 1.3E+2) = 1.9, *p* = 1.2E−1, the main effect of group: F(1, 33) = 1.8E+1, *p* = 1.4E−4, followed by Bonferroni post hoc test, day 0 (*p* = 1.0), day 4 (*p* = 1.7E−3), day 7 (*p* = 1.8E−2), day 11 (*p* = 2.9E−1), and day 14 (*p* = 8.2E−3); spine formation, the interaction effect F(4, 1.3E+2) = 7.4E−1, *p* = 5.7E−2, the main effect: F(1, 33) = 4.7, *p* = 0.50) while all spine parameters were significantly increased in the tDCS with TTX therapy mice as compared to TTX alone mice (Fig. 3f–h, TTX with tDCS vs TTX without tDCS, a two-way repeated ANOVA followed by Bonferroni post-hoc tests; spine turnover, the interaction effect: F(2.9, 81.3) = 3.7, *p* = 1.6E−2, followed by Bonferroni post hoc test, day 0 (*p* = 9.4E−1), day 3 (*p* = 9.8E−5), day 6 (*p* = 3.6E−3), day 9 (*p* = 1.7E−2), and day 12 (*p* = 1.2E−2); spine elimination, the interaction effect: F(4, 1.1E+2) = 1.7, *p* = 1.5E−1, the main effect of group: F(1, 28) = 1.5E−1, *p* = 1.0E−3; followed by Bonferroni post hoc test, day 0 (*p* = 0.81), day 3 (*p* = 1.1E−2), day 6 (*p* = 7.6E−3), day 9 (*p* = 5.0E−3), and day 12 (*p* = 6.0E−2); spine formation, the interaction effect: F(3.7, 1.0E+2) = 1.8, *p* = 0.13, the main effect: F(1, 28) = 10.0, *p* = 3.8E−3; followed by Bonferroni post hoc test, day 0 (*p* = 0.93), day 3 (*p* = 5.9E−4), day 6 (*p* = 5.8E−2), day 9 (*p* = 2.0E−1), and day 12 (*p* = 3.2E−2)). Despite these changes, the spine density was more constant during treatment, and not significantly different between hM3Dq + CNO + TTX mice and hM3Dq + saline + TTX mice (Supplementary Fig. 14e), suggesting a balance between overall spine formation and elimination. Various other control mice (mCherry + CNO + TTX; hM3Dq + CNO + vehicle; hM3Dq only) all had similar and reasonably constant rates of turnover, elimination, and formation rates during and after CNO treatment (Supplementary Fig. 15). Thus, we stratified eliminated spines into those that were present prior to PSL (pre-PSL spines), and those that appeared just after PSL (early post-PSL spines), reasoning that pre-PSL spines were more likely to represent neuronal circuits for normal somatosensory processing, whereas early post-PSL spines would be more relevant to PSL-triggered maladaptive noxious circuits. There was a significantly greater rate of loss of these post-PSL spines as compared to pre-PSL spines in CNO-treated mice, whereas spine loss rates of both pre- and post-PSL spines was similar in saline treated control mice (Fig. 4a, hM3Dq + CNO + TTX spine loss rates, pre-PSL spines: 13 ± 1.9%, early-post PSL spines: 40 ± 7.4%; hM3Dq + saline + TTX, pre-PSL spines: 6.3 ± 2.0%, early-post PSL spines: 17 ± 8.1%, a two-way ANOVA, the interaction effect: F(1, 66) = 2.2, *p* = 1.5E−1, the main effect: F(1, 66) = 1.1E+1, *p* = 2.0E−3; followed by Bonferroni post hoc test, pre-PSL vs early post-PSL in CNO-treated mice (*p* = 7.5E−4)). Closer examination of the temporal pattern of elimination of these early post-PSL spines (Fig. 4b) showed that while most were eliminated during the one week preceding treatment in both mice cohorts (“Before”), the proportion that were eliminated during the subsequent treatment period (“During”) was significantly higher in the CNO treated mice as compared to the saline controls mice (Fig. 4b, hM3Dq + CNO + TTX, before: 68 ± 4.4%, during: 14 ± 2.9%, after: 3.7 ± 1.8%, persistent: 14 ± 3.0%; hM3Dq + saline + TTX, before: 66 ± 4.7%, during: 5.9 ± 2.1%, after: 3.4 ± 1.7%, persistent: 25 ± 5.1%; hM3Dq + CNO + TTX vs hM3Dq + saline + TTX, an unpaired two-sided *t* test, Before (t(33) = 4.0E−1 *p* = 7.0E−1), During (t(33) = 2.3, *p* = 2.9E−2) and After (t(33) = 1.3, *p* =9.0E−1), Persistent (t(33) = −2.0, *p* = 5.4E−2)). When all new spines formed in the 2 weeks after PSL were grouped, 46% were subsequently lost during CNO treatment as compared to just 17% lost during saline treatment (Supplementary Fig. 14f). These results are consistent with the idea that activated astrocytes facilitate a temporary window of plasticity where dendritic spines associated with maladaptive noxious circuits (rendered inactive by afferent input blockade) are preferentially eliminated. To assess how differential elimination of these post-PSL spines may affect excitability, we quantified Ca^2+^ transients in the same S1 neuronal population in hM3Dq expressing mice subjected to PSL, before and after CNO + TTX therapy. The frequency of Ca^2+^ transients was significantly (albeit modestly) decreased, and most mice showed a decrease in averaged S1 neuronal activity (Supplementary Fig. 16). This suggests that loss of the maladaptive circuits formed after PSL may reduce the enhanced excitability of S1 subsequent to nerve injury. We propose that this preferential loss of early post-PSL spines represents a targeted pruning of the aberrant neural connections that critically contribute to the chronic tactile and thermal allodynia-like behaviour.

### Preclinical evaluation of combined tDCS and lidocaine therapy

**Fig. 5:**
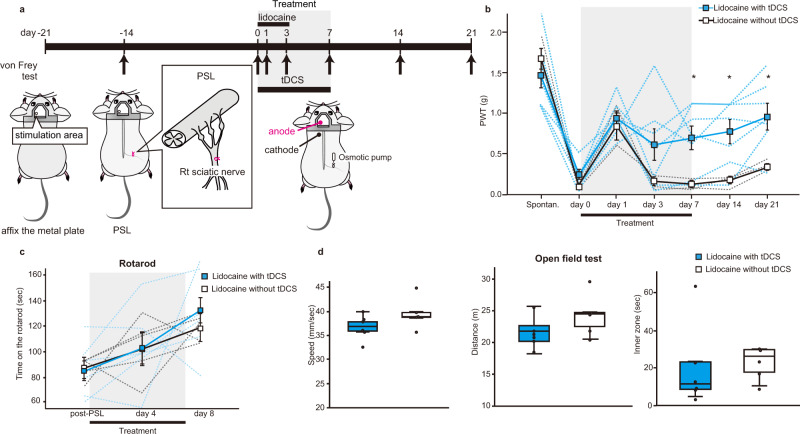
tDCS-lidocaine therapy reverses mechanical allodynia-like behaviour without affecting rotarod and open field behaviours. **a** Schematic of the experimental schedule. The start of tDCS-lidocaine (or control) treatment was designated as “day 0”. Lidocaine was continuously administered (upper black bar) to the injured right sciatic via elution from a subcutaneously implanted osmotic pump from day 0 to day 3 (when the pump was removed). Surgery for head-plate fixation and PSL, and measurement of mechanical paw withdrawal thresholds (arrows), and tDCS administration (lower black bar, day 0 to day 7) were performed as in Fig. 1. **b** Graph plotting mechanical paw withdrawal thresholds as mean ± SEM at various time-points before PSL (Spontan.), and before (day 0), during (day 1–day 7) and after (day 8 onwards) the treatment period (grey shading) for lidocaine with tDCS (blue, *n* = 7 mice) or control lidocaine-alone (white, *n* = 3 mice). Dashed-lines represent individual mice. Statistical significance was tested using a two-way repeated measures ANOVA, followed by Bonferroni post-hoc tests comparing treatment cohorts at each time-point, *p < 0.05. Source data are provided as a Source Data file. **c** Graph plotting the latency to falling during the accelerating rotarod assay at various time-points before (post-PSL), during (day 4) and after (day 8) the treatment period (grey shading) for lidocaine with tDCS (blue, *n* = 6 mice) or control lidocaine-alone (white, *n* = 5 mice). Dashed-lines represent individual mice. Statistical significance between treatment cohorts was tested using a two-way repeated measures ANOVA. Source data are provided as a Source Data file. **d** Box and whisker plots of locomotor activity (Left: speed, Centre: distance) and anxiety behaviour (Right: time spent in inner zone) following the treatment period for lidocaine with tDCS (blue, *n* = 6 mice) or control lidocaine-alone (white, *n* = 5 mice). Population medians, first and third quartiles are represented by the boxes, maxima and minima by the whiskers, and individual mice by the overlaid single points, respectively. Statistical significance between treatment cohorts was tested using an unpaired two-sided *t*-test. Source data are provided as a Source Data file.

While TTX effectively blocked noxious afferent inputs during the therapeutic window, it is too toxic to be integrated into clinical application. Therefore, we evaluated the use of a local anaesthetic, applying lidocaine via a subcutaneous osmotic pump (2 mg/kg/h) with its output aimed close to the injured sciatic nerve. Lidocaine was delivered during the 1st three days of the tDCS therapy and then the pump was surgically removed (Fig. 5a). Control mice had the same lidocaine delivery but no tDCS. While the effects of lidocaine on paw withdrawal thresholds were weaker than TTX, combined transient lidocaine with tDCS therapy still caused a sustained and significant reduction in the extent of allodynia-like behaviour that outlasted the treatment period (Fig. 5b, mean ± SEM of paw withdrawal thresholds (g); lidocaine with tDCS mice (blue, *n* = 7) were: Spontan.: 1.5 ± 0.17, day 0: 0.25 ± 6.2E−2, day 1: 0.93 ± 9.3E−1, day 3: 0.61 ± 0.19, day 7: 0.69 ± 0.15, day 14: 0.77 ± 0.15, day 21: 0.96 ± 0.16, lidocaine without tDCS mice (white, *n* = 3), Spontan.: 1.7 ± 0.13, day 0: 0.11 ± 1.6E−2, day 1: 0.86 ± 0.17, day 3: 0.16 ± 5.E−23, day 7: 0.14 ± 4.1E−2, day 14: 0.17 ± 4.2E−2, day 21: 0.33 ± 4.2E−2). The withdrawal thresholds in these two cohorts were significantly different (lidocaine with tDCS vs lidocaine without tDCS, a two-way repeated measures ANOVA: interaction effect: F(6, 48) = 2.5, *p* = 3.5E−2; followed by Bonferroni post hoc analysis, Spontan. (*p* = 0.50), day 0 (*p* = 0.18), day 1(*p* = 0.68), day 3 (*p* = 0.18), day 7 (*p* = 4.4E−2), day 14 (*p* = 3.7E−2), day 21(*p* = 4.1E−2)). As we also observed with the CNO-TTX therapy experiments (Supplementary Fig. 13), combination tDCS-lidocaine therapy did not adversely affect motor coordination in the rotarod assay (Fig. 5c, lidocaine with tDCS (blue, *n* = 6), post-PSL time to fall: 84.8 ± 9.9 s, day 4: 102.8 ± 13 s, day8: 132.4 ± 13 s; lidocaine without tDCS, *n* = 5, post-PSL time to fall: 87.2 ± 4.1 s, day 4: 102.1 ± 11 s, day8: 119.0 ± 4.6 sec; lidocaine with tDCS vs lidocaine without tDCS, a two-way repeated measures ANOVA: interaction effect: F(2, 18) = 6.9, *p* = 0.74, the main effect: F(1, 9) = 0.22, *p* = 0.65), or locomotion and anxiety-like behaviours in the open-field test (Fig. 5d, lidocaine with tDCS: (blue, *n* = 6), speed: 37 ± 1.0 mm/s, distance: 21.7 ± 1.0 m, inner zone: 20.4 ± 9.0 s; lidocaine without tDCS, (white, *n* = 5), speed: 3.9 ± 1.5 mm/s, distance: 24.4 ± 1.5 m, inner zone: 22.8 ± 3.8 s; lidocaine with tDCS vs lidocaine without tDCS unpaired two-sided *t*-test, speed (t(9) = −1.6, *p* = 0.14), distance (t(9) = −1.5, *p* = 0.16), duration in inner zone (t(9) = −0.22, *p* = 0.83)).

## Discussion

This study demonstrates that astrocytes can be positively leveraged to treat chronic pain. Specifically, transiently enhancing astrocytic Ca^2+^ activity, either through tDCS or the DREADD system, whilst simultaneously blocking noxious afferent input from the site of injury, enables increased circuit remodelling which we propose preferentially eliminates maladapted noxious circuits and thereby permanently eliminates mechanical allodynia and returns tactile sensation to normal. This is a conceptual shift in chronic pain treatment, from targeting neuronal excitability and synaptic transmission that reduces the consequences of hyperactive circuits, to targeting astrocytes and cortical circuit plasticity, which may actually reverse some of the mechanisms by which acute pain transitions to chronic pain.

Our study provides further support for the growing idea that aberrent neural circuit plasticity in S1 significantly underpins intractable nociceptive perceptions in chronic pain^11,21^. Hyperactivity of L2/3^21^, loss of effective local circuit inhibiton^22^, and activation of astrocytes to mediate spine plasticity^11,20^ have all been observed during early chronic pain development in rodent models and prophylactically reducing cortical neuronal and astrocyte activity has been shown to prevent robust chronic pain development. Our current report shows that targeting S1 circuits weeks after allodynia has developed can still ameliorate pain symptoms and reverse the injury-associated S1 plasticity. We propose that the tDCS-TTX treatment strategy reduces connectivity in S1 noxious circuits. Other spinal and brain regions are undoubtedly important for chronic pain and its complex behavioural and neural components. In particular, neural activity in the insular and anterior cingulate cortices are associated with the more affective and emotional aspects of pain and interact with both S1 and the secondary somatosensory cortices and extensive other brain regions in the processing of pain^23,24^. We do not yet know how our therapy may impact on other pain regions and their behavioural correlates, and our current work is restricted to the more sensory discriminative aspects of neuropathic pain.

A key component of this therapeutic strategy was the concurrent application of TTX or lidocaine. We believe the doses and delivery systems used fortuitously and selectively reduced noxious afferent input without large impacts on tactile and motor fibres. A reduction of afferent input can very effectively weaken the synaptic representation of those afferents in cortical areas^25^. We propose reduction in nociceptive afferent activity by TTX or lidocaine weakens the maladapted cortical circuits responding to the painful tactile stimuli, exposing these weakened synapses to be eliminated, by astrocyte or microglial phagocytosis^26,27^. Because some tactile sensation remains, circuits responding to “normal” sensation can be strengthened, returning S1 representations and activity back to control. Concurrent astrocyte activity may either facilitate synapse removal, or strengthen rewiring of “normal” circuits^28,29^. The current clinical use of nerve block approaches in neuropathic pain similarly targets excessive nociceptive efferent activity^30^ whilst sparing tactile and motor function, and hence may be suitable to complement tDCS to selectively weaken aberrant maladapted cortical noxious circuits.

Our study and that of Monai et al.^15^ indicates that cortical astrocytes are activated by lower tDCS current stimulation levels (0.1 mA or less) than required to activate cortical pyramidal neurons. Indeed a direct study applying tDCS to rodents while recording membrane potential and spiking patterns reported that currents of over 0.4 mA were required to influence cortical membrane potential and network oscillations^31^. How astrocytes are activated without inducing marked transients in cortical neuronal soma is intriguing. Monai et al.^15^ showed a role of adrenergic A1 receptor activation in tDCS-induced astrocyte activation, and suggested noradrenaline was released during tDCS from locus coeruleus neurons and/or from the activation of cortical nerve terminals. Further insights into signalling pathways mediating astrocyte activation by tDCS and the subsequent events mediating the proposed rewiring of neural circuits may reveal additional therapeutic targets for chronic pain.

Our study suggests combined tDCS and afferent nerve blockade warrants further clinical investigation as a therapy for pain. tDCS is being widely trialled for different neurocognitive applications, particularly depression, stroke recovery and chronic pain, and at typical current intensities is considered safe^12^. Based on our results in mice, we would envisage thrice daily stimulation over one week with the anode placed over the S1 approximate to the injured region, and using a more modest stimulation intensity to preferentially activate astrocytes rather than neurons. Regional peripheral nerve block via a catheter implanted under ultrasound-guided surgery is used commonly for postoperative analgesia^32,33^ and we would also envisage concurrent application of a long-lasting local anaesthetic bolus via this route. Alternatively, continuous infusion via a controlled pump for 4–6 days has also been trialled to treat different chronic pain syndromes^34,35^. However, it should be recognized that our current study only used young male mice, many pain phenotypes differ between sexes with females typically reporting greater pain responses^36,37^. A further limitation of our study regards translating efficacy from a single pain model in one mouse strain to humans; for example, pregabalin has larger and broader analgesic effects in rodent chronic pain models than observed clinically^38^.

In conclusion, we report a transient combination therapy that reverses mechanical allodynia in mice. We propose that activating cortical astrocytes while reducing peripheral noxious inputs engineer synaptic plasticity that breaks down inappropriate neural connections formed during the transition from acute to chronic pain. Given that both nerve block and tDCS are both readily utilized in the clinic, it seems feasible to translate our discovery into clinical practice for the treatment of intractable chronic pain associated with a well defined peripheral injury.

## Methods

### Care and use of animals

All animal experiments were approved by the Institutional Animal Care and Use Committee of the National Institutes for Natural Sciences, Okazaki, Japan. Every effort was made to minimize the suffering and number of animals used.

All animal experiments were conducted using male, 8–10 week old C57BL/6 mice (Centre for Animal Resources and Collaborative Study, National Institutes of Natural Sciences and Japan SLC), M-line mice (C57BL/6 mice expressing enhanced green fluorescent protein (EGFP) in sparse subsets of cortical neurons under the thy-1 promoter, Centre for Animal Resources and Collaborative Study, National Institutes of Natural Sciences)^39^ or mGFAP-Cre mice (C57BL/6 mice expressing Cre recombinase in astrocytes under the mouse GFAP promoter (astrocyte specific promoter), Centre for Animal Resources and Collaborative Study, National Institutes of Natural Sciences)^40^. All mice were housed with ad libitum access to standard rodent chow and water under a 12 h light-dark cycle. Mice were randomly separated to treatment or control groups.

### Partial sciatic nerve ligation (PSL) and behavioural testing

The PSL model is a well-established animal model of chronic pain that is characterized by protracted touch-evoked allodynia-like behaviour and hyperalgesia-like behaviour^16^. PSL was performed by ligating the dorsal one-third to half of the right sciatic nerve with 8–0 silk suture under 1.5–1.8% isoflurane anaesthesia.

Mechanical-tactile allodynia-behaviour was observed for 2 weeks post-PSL before further experiments. Mechanical allodynia-like behaviour and hyperalgesia were assessed manually using the von Frey filament test. All testing was performed during the 12 h daylight cycle. Prior to von Frey testing, mice were habituated individually for 30 min in the testing chamber which consisted of a small plastic cage with a mesh floor. The von Frey filaments were applied through the mesh floor so as to perpendicularly touch the hind paw with sufficient force to cause slight filament buckling. If this elicited withdrawal, licking or shaking of the paw by the mice, a positive response (pain-like behaviour) was recorded. Filaments were tested with increasing then decreasing force to establish that which caused a median 50% paw withdrawal threshold^41^. All von Frey testing was performed in a blinded fashion with the experimenter unaware of the treatments the mice had received. There were no differences in the baseline thresholds for mechanical sensitivity between the three strains of mice used (C57BL/6, M-line and mGFAP-Cre).

Thermal sensitivity was assessed following injury (post-PSL) and after treatment (at days 8 and 14) using the Plantar test^42^ with an infrared heat stimulus and an automated recording of latency to paw withdrawal (Hargreaves Apparatus, Ugo Basile, Italy). As previously described^43^, mice were placed in the thermal sensitivity enclosure for 60 min for each of 2 days prior to testing to habituate to the plantar test environment. When mice positioned their hind paw on the infrared emitter/detector, heat stimulation started and continued until the paw was withdrawn. The latency to paw withdrawal was observed over 5 trials, with the averaged value excluding the two trials with the smallest and largest withdrawal times.

The accelerating rotarod test was performed to assess motor coordination as previously described^44,45^. Briefly, mice were habituated to the rotarod (LE8200, Panlab, Spain) over 30 min on two consecutive days prior to the trial. For the test trial, the rotation frequency began at 4 rpm and accelerated to 40 rpm over 5 min with the time at which mice fell from the rotarod noted. The rotarod test was repeated twice at 20 min intervals, with the latencies to fall averaged from both trials.

The open field test was performed to assess locomotor activity and anxiety-like behaviour. As previously described^46^, mice were placed in a 40 × 40 cm chamber and exploratory behaviours recorded for 10 min and subsequently analyzed using an automated tracking system (Lab Squirred Pty Ltd, Sydney, Australia). The open field was divided into an inner (20 × 20 cm) and outer zone, to assess anxiety-like behaviour. We measured total locomotion speed, total distance travelled, and the total duration spent in the inner zone.

### Preparation and implantation of TTX mixed with Elvax, and of the lidocaine osmotic pump

As described previously^47^, TTX was applied to the right sciatic nerve using an ethylene-vinyl acetate copolymer (Elvax) carrier. Briefly, 100 mg/mL Elvax solution was prepared by dissolving Elvax beads (Dupont) in dichloromethane, before adding 3 mM TTX to give a final TTX concentration of 300 μM. This TTX-Elvax solution was then stirred for 1 h to ensure thorough mixing before pouring onto a pre-cooled glass dish, which had been incubated for 2–3 h at −80 °C, and stored at −20 °C. This allowed for the complete evaporation of the dichloromethane and for the TTX-Elvax solution to solidify into a sheet that could then be applied around the right sciatic nerve just proximal to the PSL injury (under 1.5–1.8% isoflurane anaesthesia). The estimated duration for drug delivery using Elvax is 5–6 days^11^.

Lidocaine (2 mg/kg/h in saline) was applied using an osmotic pump (0.5 μl/h, 1007D, Alzet, CA) implanted subcutaneously in the back with the tip of the osmotic pump implanted close to the sciatic nerve ligation. After 3 days, the osmotic pump was removed. Implantation and removal surgery occurred under isoflurane anaesthesia.

### Generation of adeno-associated viruses

pAAV-gfaABC1D-mCherry was prepared by generating a linearized vector from pAAV-gfaABC1D-hM3D(Gq)-mCherry (Addgene, #50478) using restriction enzymes with subsequent vector end blunting and nick sealing mediated by DNA polymerase I Large (Klenow) Fragment and T4 DNA ligase respectively. pAAV-gfaABC1D-GCaMP6f was prepared by incorporating the GCaMP6f sequence from pGP-CMV-GCaMP6f (Addgene, #40755) into a pAAV-gfaABC1D- vector (Addgene, #50478)^48^ using the In-Fusion cloning kit (Takara, Mountain View, CA) and restriction enzymes. All plasmids sequences were confirmed by direct-PCR.

Adeno-associated viruses (AAV’s) were generated as described previously^49^. Briefly, HEK293 cells were transfected with pAAV-GFAP-mCherry or pAAV-GFAP-GCaMP6f, pHelper (Cell Biolabs, Inc. San Diego, CA), and pRC5 (Cell Biolabs, Inc. San Diego, CA). The surrounding media from each culture plate was collected 3 days later and purified for AAV particles using a tangential flow filter system (VIVAFlow50 and Masterflex Economy Drive Peristaltic Pump, Sartorius, Germany). The dialyzed product was concentrated using an Amicon Ultra-15 filter (MWCO, 100 kDa, Merck, Germany) before benzonase (Merck, Germany) was added at 100 units per culture plate, then PBS was added and the mixture incubated at 37 °C for 30 min. The incubated mixture was then transferred into an ultracentrifuge tube, forming the top solution layer over an iodixanol solution series of increasing concentration (8 ml of 15%, 6 ml of 25%, 5 ml of 40%, and 5 ml of 60%; top to bottom). After 3 h of ultracentrifugation at 230,000 × *g* and 18 °C, the 40% iodixanol fraction was aspirated using a 18-gauge needled syringe and concentrated using an Amicon Ultra-15 filter to give the finished product. Virus titres were measured by quantitative real-time PCR analysis (Thunderbird SYBR qPCR Mix, TOYOBO CO., Osaka, Japan).

AAV2/5-gfaABC1D-hM3D(Gq)-mCherry generation from pAAV-gfaABC1D-hM3D(Gq)-mCherry (Addgene, #50478) was outsourced for AAV custom vector production (UNC vector core, NC, USA).

### AAV injection

Mice used for the DREADD-behaviour only experiments were injected with AAV2/5-gfaABC1D-hM3D(Gq)-mCherry as follows, 1–2 weeks prior to the PSL operation. Mice were anesthetized (i.p.) using a ketamine (70 mg/kg) and xylazine (10.5 mg/kg) mixture and mounted into a stereotaxic frame (NARISHIGE, Japan). Approximately 500 nL of AAV was injected into the left S1 at stereotaxic coordinates 0.5 mm posterior and 1.5 mm lateral to Bregma, and at a depth of 300 μm below the brain surface. The injection pipettes were pulled from filament-containing glass capillaries (GDC-1, NARISHIGE, Japan) and AAV slowly expelled (over 10 min) using pneumatic pressure (IM 300 Microinjector, Narishige Scientific Instrument Lab., Tokyo, Japan).

### Immunohistochemistry

Brains for GFAP immunohistochemistry were fixed by cardiac perfusion with 4.0% paraformaldehyde (Muto Pure Chemicals Co., Tokyo, Japan) under anaesthesia (i.p.) using ketamine (70 mg/kg) and xylazine (10.5 mg/kg) on day 4 of the CNO treatment protocol (2 h after 12 repeated CNO injections). Following overnight post-fixation in 4.0% paraformaldehyde, the fixed brains were cut using a vibratome (VT1006S, Leica, Germany) into 50 μm thick sections. These were incubated over 2 nights at 4 °C with primary antibodies – rabbit anti-S100β (1:500; EP1576Y, ab52642, Abcam, Cambridge, UK), mouse anti-NeuN (1:400; 1B7, AB104224, Abcam, Cambridge, UK), or rabbit anti-GFAP (1:500; ab5804, Merck, Germany). Immunocomplexes were visualized by chemiluminescent detection using goat anti-rabbit/anti-mouse secondary antibodies (1:300; Santa Cruz Biotechnology). Three images for each mouse were acquired using an A1R confocal microscope (Nikon, Tokyo, Japan) with NIS-elements software (Nikon, Tokyo, Japan) under a ×20 objective lens (PLanSApo, NA = 0.75) with a z-step size of 0.5 μm. To detect hM3Dq-mCherry expression, brains were fixed at 4 weeks after the corresponding AAV injection. The total number of mCherry-expressing cells in these S1 was manually counted using the Cell Counter plugin (credit Durt De Vos) within the ImageJ software environment (NIH) and expressed relative to the total number of cells stained with S100β or NeuN.

### Open-skull chronic cranial window implantation

Mice used for in vivo imaging were injected with the relevant AAV’s and implanted with a chronic cranial window according to the following procedure. The cranial window surgery was undertaken two weeks prior to the PSL operation.

Mice were anesthetized (i.p.) using a ketamine (70 mg/kg) and xylazine (10.5 mg/kg) mixture. The scalp skin was incised and the entire skull surface was waterproofed with tissue adhesive (3 M Vetbond, 3 M, MN, USA). A custom-made metal head plate was then directly attached to the skull using resin cement (Estecem II, Tokuyama Dental Corporation, Tokyo, Japan) and dental cement (Fuji Lute BC, GC Corporation, Tokyo, Japan). After the cements had cured, the entire skull surface was covered with dental adhesive (Super-Bond with Catalyst V, Monomer and Polymer, Sun Medical Corporation, Shiga, Japan) which acted as both a reinforcing and waterproofing agent.

Implantation of the cranial window was performed the next day. Mice were anaesthetized with 1.0–1.2% isoflurane and secured in a stereotaxic frame via the attached head plate. For S1 imaging, a circular 2–3 mm diameter craniotomy was then drilled over the hind-limb area of the left S1 (craniotomy centre at 0.5 mm posterior and 1.5 mm lateral to Bregma). Following this, approximately 500 nL of AAVs (gfaABC1D-hM3Dq-mCherry, gfaABC1D-GCaMP6f, gfaABC1D-mCherry, and CAG-flex-GCaMP6f (UPENN)) were injected as described above into the centre of the craniotomy site at a depth of 300 µm from the brain surface. Comparative M1 and S1 imaging used a single rectangular (3 × 2 mm) craniotomy through which approximately 500 nL of AAVs (CamKII-GCaMP6f (UPENN), gfaABC1D-hM3Dq-mCherry, gfaABC1D-GCaMP6f) were injected into M1 and S1 areas at a depth of 300 µm from the brain surface (Supplementary Fig. 9a). The craniotomy sites were covered with a double glass coverslip (Matsunami Glass, Osaka, Japan) which consisted of a 2 mm diameter coverslip fused to a 4.5 mm diameter coverslip, or a 2 × 3 mm coverslip fused to a 4 × 5 mm coverslip. The coverslip was fixed to the skull using a mixture of dental cement (Fuji Lute BC, GC Corporation, Tokyo, Japan) and dental adhesive (Super-Bond with Catalyst V, Monomer and Polymer, Sun Medical Corporation, Shiga, Japan).

### Ca^2+^ imaging of astrocytes and neurons in S1 or M1 for DREADD and tDCS assessment

Ca^2+^ imaging was performed 2 weeks after cranial window implantation using a multiphoton microscope (Nikon A1R MP, Nikon, Tokyo, Japan) fitted with a 25× water immersion objective lens (Nikon Apo LWD 25×/1.10w, Nikon, Tokyo, Japan) and a Ti:Sapphire laser (MaiTai DeepSee, Spectra Physics, CA, USA), tuned to 950 nm for 2-photon excitation of GCaMP6f.

During the imaging session, mice were secured in a custom-built frame via their surgically attached head plate. mGFAP-Cre and C57BL6/J mice anaesthetized with isoflurane (2% for induction, 1% for maintenance) were used to assess CNO-induced Ca^2+^ activity. Awake C57BL6/J mice were used to assess tDCS-induced Ca^2+^ activity, after habituation to the head fixation frame for 5 days prior to imaging session by daily fixing to the head frame for 15 min, during which time there was free access to water. To determine neural activity, Ca^2+^ fluorescence was recorded from the soma of layer 5 pyramidal neurons in mice freely moving on a moveable running baseplate while head-fixed^50^. Mice were further habituated to the running baseplate during fixation with 10 min sessions over 2 days prior to imaging.

Astrocyte regions of interest (ROIs) were detected semi-automatically using the GECIquant plugin^51^ within the ImageJ software environment (NIH). The ROIs corresponding to neurons were detected using a previously described MATLAB script^52,53^. The change in the Ca^2+^ fluorescence within detected ROIs was subsequently analyzed using MATLAB. Baseline mean fluorescence (*F*_0_) was defined as the mean intensity of the first 50 frames acquired before tDCS or CNO administration. The difference between the observed fluorescence over a given period of time (*F*) and *F*_0_ was then calculated and normalized against *F*_0_ to give the change in Ca^2+^ fluorescence signal amplitude (Δ*F/F*_0,_ where Δ*F* = *F* *−* *F*_0_). Ca^2+^ events were defined as periods where the Δ*F/F*_0_ values in a single ROI exceeded a *F*_0_ + 3*SD threshold. Quantitative comparison of Δ*F/F*_0_ between mice was performed using area under the curve values (AUCs). Neuronal activity during locomotion was quantified by the number of Ca^2+^ transient spikes per image frame (20 min, with spikes determined using a threshold of *F*_0_ + 4*SD)^50^. The number of Ca^2+^ transient spikes were calculated by integrating the area of each Ca^2+^ event for every ROI in the field of view and summing these together^54^.

### Transcranial direct current stimulation (tDCS)

tDCS has traditionally been used to non-invasively modulate neuronal activity. In this study, however, we used a tDCS protocol to selectively activate astrocytes^15^. In mice used for tDCS-behaviour alone experiments, PSL was performed 1 week after attaching the metal head plate but 2 weeks before preparing the skull for tDCS stimulation. In mice used for tDCS-imaging experiments, cranial window implantation and AAV injection were performed 1 day after attaching a metal head plate and 2 weeks before preparation for tDCS. PSL was not performed in these mice.

To prepare for tDCS, mice were anesthetized (i.p.) using a ketamine (70 mg/kg) and xylazine (10.5 mg/kg) mixture. The scalp skin was incised, and the entire skull surface was waterproofed with a tissue adhesive (3 M Vetbond, 3 M, MN, USA). A custom-made metal head plate was then directly attached to the skull using resin cement (Estecem II, Tokuyama Dental Corporation, Tokyo, Japan) and dental cement (Fuji Lute BC, GC Corporation, Tokyo, Japan). After the cements had cured, the entire skull surface was covered with dental adhesive (Super-Bond with Catalyst V, Monomer and Polymer, Sun Medical Corporation, Shiga, Japan) which acted as both a reinforcing and waterproofing agent.

On the first day of tDCS therapy (day 0), the cathode and anode sites for tDCS were prepared immediately after performing PSL von Frey testing. For the cathode, a 0.5 mm diameter silver wire (The Nilaco corporation, Tokyo, Japan) was subcutaneously and permanently implanted in the neck. For the anode, the dental adhesive was removed from a 5 mm^2^ diameter circular area of the skull 2 mm posterior to the left S1 (Supplementary Fig. 1). During each tDCS session, a silver wire was placed within this anode skull site which was filled with conductive gel (Gelaid, Nihon Kohden, Japan). Between tDCS sessions, the wire was removed and this anode skull site was covered with silicon to keep the skull clean and moist.

tDCS therapy consisted of ten-minute individual sessions performed at 8-hourly intervals over 1 week (21 tDCS sessions in total). PSL von Frey testing at day 0 was followed 2–4 h later by the first tDCS session. Subsequent von Frey testing at days 1, 3, 5, and 7 were performed 2 h after the third, 9th, 15th, and 21st tDCS session, respectively. During each tDCS session, a constant 0.1 mA of direct current was applied from the anode to the cathode over the 10 min using a stimulus isolator (Nihon Kohden, Japan) (Supplementary Fig. 1). During this time, mice were awake but secured in a custom-made frame via their attached head plate. TTX without tDCS mice were subjected to the same procedure but without any current stimulation. In experiments where the effect of tDCS parameters on astrocytic or neuronal activity was assessed by simultaneous imaging, the constant current varied between each session from 0.01 to 0.1 mA for astrocytes and from between 0.01 and 1.0 mA for neurons.

To block astrocyte activation, a small sheet of Elvax mixed with fluoroacetate (astrocytic Krebs cycle inhibitor) was applied directly onto the S1 cortex as previous described^17^, and after von Frey test on day 0. For Elvax preparation, Elvax beads (20 mg) were dissolved in 26 mM fluoroacetate and 200 μl dichloromethane by stirring with a voltex for 1 h. Then, the Elvax solution was plated on a glass dish and kept at −80 °C for 2 h. The Elvax sheet was cut into 1 mm squares before use. Following Elvax application, the open skull area was covered with silicon.

### Long-term in vivo 2-photon imaging of spine dynamics

In these experiments, M-line mice were injected with the relevant AAV’s and implanted with a chronic cranial window as described above. The first imaging session was performed 14–20 days after cranial window implantation and thus preceded the PSL operation by 3 days. Subsequent imaging sessions were performed at 14, 10, and 7 days prior to the first CNO injection (day 0) and on days 0, 4, 7, 11, and 14 (Fig. 3a).

During each imaging session, mice were anesthetized using 1.0–1.5% isoflurane. The imaging area position was determined as previously described^11^ by using intrinsic optical signal imaging and identifying the mCherry expressing area. In vivo 2-photon imaging was performed using a multiphoton microscope fitted with a 25× water immersion objective lens and a Ti:Sapphire laser tuned to 800 and 950 nm for 2-photon excitation of mCherry and EGFP, respectively. In order to perform reliable long-term imaging of the same specific layer 5 pyramidal neuron dendrite over multiple imaging sessions, the dendritic area of interest was first identified using low-magnification imaging (512 × 512 pixels, 0.99 μm/pixel, 2 μm z step). High-magnification (512 × 512 pixels, 0.12 μm/pixel, 0.5 μm z step) was then used to quantify the specific dendritic region morphology.

For analysis, all images were first imported into the ImageJ software environment (NIH) in order to create 3D image stacks for each dendrite, with motion artefacts corrected using TurboReg. The 3D image stacks were then imported into the AIVIA software environment (DRvision Technologies LLC, DC, USA) which was able to automatically identify and track individual spines on the same section of dendrite across all imaging sessions (with manual cross-checking). All types of dendritic protrusions were included for analysis, except for those which were located in close proximity to the distal tip of a dendritic branch, due to previous reports of their significantly greater instability^11^. Spine parameters were quantified by other experimenters who did not perform therapy or spine imaging, and were blinded to the treatment/control group.

When comparing 2 successive imaging sessions, spines that were present in the latter session but not the former session were referred to as “formed” spines whereas spines that were present in the former session but not in a latter session were referred to as “lost” spines. The spine formation rate between 2 successive imaging sessions was calculated as the number of formed spines divided by the total number of spines counted in the former session. The spine elimination rate between 2 successive imaging sessions was calculated as the number of lost spines divided by the total number of spines counted in the former session. The spine turnover rate between 2 successive imaging sessions was an average of these values—calculated as the sum of the number of formed spines and lost spines, divided by twice the total number of spines counted in the former session. Spine density was calculated as the total number of spines counted, divided by the length of the dendrite. Spine densities were normalized to that counted on day 0.

### Statistical analysis

All statistical analyses were performed using SPSSver22 (IBM, NY, USA) or MATLAB (The Mathworks, Inc). Parametric or non-parametric statistical tests were selected by determining first if the data was normally distributed using the Kolmogorov–Smirnov normality test. Comparisons between different sample groups within the same treatment cohort were performed using a two-way repeated ANOVA followed by the Bonferroni post hoc test. Non-parametric multiple comparisons between different treatment cohorts were performed using the Kruskal–Wallis H test followed by the Bonferroni post-hoc test. Comparisons between different treatment cohorts were performed using the Chi-squared test. Single variable comparisons were performed using paired, unpaired two-sided *t*-tests or Wilcoxon rank sum test following confirming a normal distribution or not using Kolmogorov–Smirnov normality test. Values of *p* < 0.05 were considered to be statistically significant. All values were reported as mean ± standard error.

### Reporting summary

Further information on research design is available in the Nature Research Reporting Summary linked to this article.

## Supplementary information

Supplementary Information

Reporting Summary

## Data Availability

All data associated with this study are provided in the Supplementary Information/Source Data file. Source data are provided with this paper.

## Source data

Source Data

## References

[1] Dahlhamer J (2018). Prevalence of chronic pain and high-impact chronic pain among adults—United States, 2016. MMWR Morb. Mortal. Wkly. Rep..

[2] Price TJ (2018). Transition to chronic pain: Opportunities for novel therapeutics. Nat. Rev. Neurosci..

[3] Woolf CJ (1983). Evidence for a central component of post-injury pain hypersensitivity. Nature.

[4] Basbaum AI, Bautista DM, Scherrer G, Julius D (2009). Cellular and molecular mechanisms of pain. Cell.

[5] Latremoliere A, Woolf CJ (2009). Central sensitization: A generator of pain hypersensitivity by central neural plasticity. J. Pain..

[6] Bushnell MC (1999). Pain perception: Is there a role for primary somatosensory cortex. Proc. Natl Acad. Sci. USA.

[7] Birbaumer N (1997). Effects of regional anesthesia on phantom limb pain are mirrored in changes in cortical reorganization. J. Neurosci..

[8] Flor H (2003). Remapping somatosensory cortex after injury. Adv. Neurol..

[9] Clarke LE, Barres BA (2013). Emerging roles of astrocytes in neural circuit development. Nat. Rev. Neurosci..

[10] Chung WS, Allen NJ, Eroglu C (2015). Astrocytes control synapse formation, function, and elimination. Cold Spring Harb. Perspect. Biol..

[11] Kim SK, Nabekura J (2011). Rapid synaptic remodeling in the adult somatosensory cortex following peripheral nerve injury and its association with neuropathic pain. J. Neurosci..

[12] Bikson M (2016). Safety of transcranial direct current stimulation: Evidence based update 2016. Brain Stimul..

[13] Jensen MP, Day MA, Miro J (2014). Neuromodulatory treatments for chronic pain: Efficacy and mechanisms. Nat. Rev. Neurol..

[14] O’Connell NE, Marston L, Spencer S, DeSouza LH, Wand BM (2018). Non-invasive brain stimulation techniques for chronic pain. Cochrane Database Syst. Rev..

[15] Monai H (2016). Calcium imaging reveals glial involvement in transcranial direct current stimulation-induced plasticity in mouse brain. Nat. Commun..

[16] Seltzer Z, Dubner R, Shir Y (1990). A novel behavioral model of neuropathic pain disorders produced in rats by partial sciatic nerve injury. Pain.

[17] Ishikawa T (2018). Cortical astrocytes prime the induction of spine plasticity and mirror image pain. Pain.

[18] Roth BL (2016). DREADDs for neuroscientists. Neuron.

[19] Wess J, Nakajima K, Jain S (2013). Novel designer receptors to probe GPCR signaling and physiology. Trends Pharmacol. Sci..

[20] Kim SK (2016). Cortical astrocytes rewire somatosensory cortical circuits for peripheral neuropathic pain. J. Clin. Invest..

[21] Eto K (2011). Inter-regional contribution of enhanced activity of the primary somatosensory cortex to the anterior cingulate cortex accelerates chronic pain behavior. J. Neurosci..

[22] Cichon J, Blanck TJJ, Gan WB, Yang G (2017). Activation of cortical somatostatin interneurons prevents the development of neuropathic pain. Nat. Neurosci..

[23] Xiao X, Zhang YQ (2018). A new perspective on the anterior cingulate cortex and affective pain. Neurosci. Biobehav Rev..

[24] Lu C (2016). Insular cortex is critical for the perception, modulation, and chronification of pain. Neurosci. Bull..

[25] Rittenhouse CD, Shouval HZ, Paradiso MA, Bear MF (1999). Monocular deprivation induces homosynaptic long-term depression in visual cortex. Nature.

[26] Schafer DP (2012). Microglia sculpt postnatal neural circuits in an activity and complement-dependent manner. Neuron.

[27] Chung WS (2013). Astrocytes mediate synapse elimination through MEGF10 and MERTK pathways. Nature.

[28] Allen NJ, Eroglu C (2017). Cell biology of astrocyte-synapse interactions. Neuron.

[29] Di Castro MA (2011). Local Ca2+ detection and modulation of synaptic release by astrocytes. Nat. Neurosci..

[30] Practice guidelines for chronic pain management: An updated report by the American Society of Anesthesiologists Task Force on Chronic Pain Management and the American Society of Regional Anesthesia and Pain Medicine. *Anesthesiology* **112**, 810–833 (2010). 10.1097/ALN.0b013e3181c43103 20124882

[31] Vöröslakos M (2018). Direct effects of transcranial electric stimulation on brain circuits in rats and humans. Nat. Commun..

[32] Ilfeld BM (2017). Continuous peripheral nerve blocks: An update of the published evidence and comparison with novel, alternative analgesic modalities. Anesth. Analg..

[33] Ilfeld BM (2011). Continuous peripheral nerve blocks: A review of the published evidence. Anesth. Analg..

[34] Ilfeld BM (2013). Liposome bupivacaine in peripheral nerve blocks and epidural injections to manage postoperative pain. Expert Opin. Pharmacother..

[35] Dadure C, Capdevila X (2005). Continuous peripheral nerve blocks in children. Best. Pract. Res. Clin. Anaesthesiol..

[36] Mogil JS (2020). Qualitative sex differences in pain processing: Emerging evidence of a biased literature. Nat. Rev. Neurosci..

[37] Muralidharan A, Sotocinal SG, Austin JS, Mogil JS (2020). The influence of aging and duration of nerve injury on the antiallodynic efficacy of analgesics in laboratory mice. Pain. Rep..

[38] Federico CA, Mogil JS, Ramsay T, Fergusson DA, Kimmelman J (2020). A systematic review and meta-analysis of pregabalin preclinical studies. Pain.

[39] Feng G (2000). Imaging neuronal subsets in transgenic mice expressing multiple spectral variants of GFP. Neuron.

[40] Garcia AD, Doan NB, Imura T, Bush TG, Sofroniew MV (2004). GFAP-expressing progenitors are the principal source of constitutive neurogenesis in adult mouse forebrain. Nat. Neurosci..

[41] Chaplan SR, Bach FW, Pogrel JW, Chung JM, Yaksh TL (1994). Quantitative assessment of tactile allodynia in the rat paw. J. Neurosci. Methods.

[42] Hargreaves K, Dubner R, Brown F, Flores C, Joris J (1988). A new and sensitive method for measuring thermal nociception in cutaneous hyperalgesia. Pain.

[43] Cheah, M., Fawcett, J. W. M. C. & Andrews, M. R. Assessment of thermal pain sensation in rats and mice using the Hargreaves test. *Bio. Protoc*. 10.21769/BioProtoc.2506 (2017). 10.21769/BioProtoc.2506 PMC5600253 28920069

[44] Kakegawa W (2011). D-serine regulates cerebellar LTD and motor coordination through the δ2 glutamate receptor. Nat. Neurosci..

[45] Rothwell PE (2014). Autism-associated neuroligin-3 mutations commonly impair striatal circuits to boost repetitive behaviors. Cell.

[46] Seibenhener, M. L. & Wooten, M. C. Use of the open field maze to measure locomotor and anxiety-like behavior in mice. *J. Vis. Exp*. 10.3791/52434 (2015). 10.3791/52434 PMC4354627 25742564

[47] Kakizawa S (2005). Maintenance of presynaptic function by AMPA receptor-mediated excitatory postsynaptic activity in adult brain. Proc. Natl Acad. Sci. USA.

[48] Lee Y, Messing A, Su M, Brenner M (2008). GFAP promoter elements required for region-specific and astrocyte-specific expression. Glia.

[49] Strobel B, Miller FD, Rist W, Lamla T (2015). Comparative analysis of cesium chloride- and iodixanol-based purification of recombinant adeno-associated viral vectors for preclinical applications. Hum. Gene Ther. Methods.

[50] Inagaki S (2019). Imaging local brain activity of multiple freely moving mice sharing the same environment. Sci. Rep..

[51] Srinivasan R (2015). Ca(2+) signaling in astrocytes from Ip3r2(-/-) mice in brain slices and during startle responses in vivo. Nat. Neurosci..

[52] Pnevmatikakis EA (2016). Simultaneous denoising, deconvolution, and demixing of calcium imaging data. Neuron.

[53] Agetsuma M, Hamm JP, Tao K, Fujisawa S, Yuste R (2018). Parvalbumin-positive interneurons regulate neuronal ensembles in visual cortex. Cereb. Cortex.

[54] Reznichenko L (2012). In vivo alterations in calcium buffering capacity in transgenic mouse model of synucleinopathy. J. Neurosci..

