# Supplementary material for: Controlled activation of cortical astrocytes modulates neuropathic pain-like behaviour
Source: Nat Commun. 2022 Jul 14;13:4100. doi: 10.1038/s41467-022-31773-8 (PMC9283422; doi:10.1038/s41467-022-31773-8)
Supplement: Supplementary file 1 — Supplementary Information [file 41467_2022_31773_MOESM1_ESM.pdf]

SUPPLEMENTAL INFORMATION TITLES AND LEGENDS

Controlled activation of cortical astrocytes modulates neuropathic pain-like behaviour

Ikuko Takeda[1,2,3], Kohei Yoshihara[4], Dennis L. Cheung[1], Tomoko Kobayashi[1], Masakazu Agetsuma[1,5], Makoto Tsuda[4], Kei Eto[1,6], Schuichi Koizumi[7,8], Hiroaki Wake[2,3,9], Andrew J. Moorhouse[10], Junichi Nabekura[1,11,12]*

Affiliations

[1]Division of Homeostatic Development, National Institute for Physiological Sciences, Okazaki, Japan**.**

[2]Department of Anatomy and Molecular Cell Biology Graduate School of Medicine, Nagoya University, Nagoya, Japan.

[3]Division of Multicellular Circuit Dynamics, National Institute for Physiological Sciences, Okazaki, Japan.

[4]Department of Molecular and System Pharmacology, Graduate School of Pharmaceutical Sciences, Kyushu University, Fukuoka, Japan.

[5]Division of Molecular Design, Research Center for Systems Immunology, Medical Institute of Bioregulation, Kyushu University, Fukuoka, Japan.

[6]Department of Physiology, School of Allied Health Sciences, Kitasato University, Sagamihara, Kanagawa, Japan.

[7]Department of Neuropharmacology, Interdisciplinary Graduate School of Medicine, University of Yamanashi, Yamanashi, Japan.

[8]GLIA Center, University of Yamanashi, Yamanashi, Japan.

[9]Center of Optical Scattering Image Science Department of Systems Science, Kobe University, Kobe, Japan.

[10]Department of Physiology, School of Medical Sciences, The University of New South Wales, Sydney, Australia.

[11]Graduate School of Medicine, Nagoya University, Nagoya, Japan.

[12] Department of Physiological Sciences, Graduate University for Advanced Studies, SOKENDAI, Hayama, Japan

***Corresponding author. Email:** nabekura@nips.ac.jp

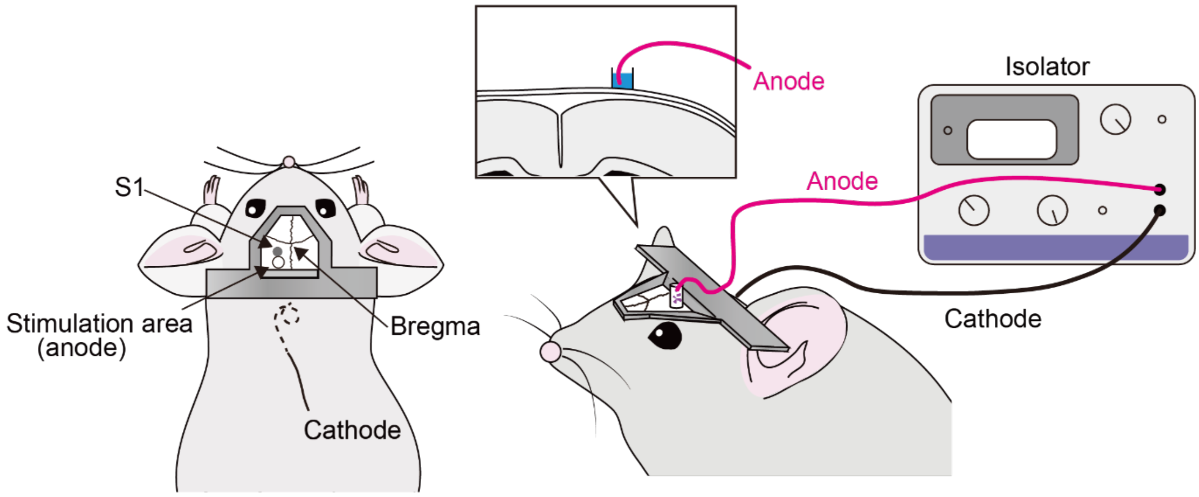

Supplementary Fig. 1. Schematic of the transcranial direct current stimulation (tDCS) setup. For the tDCS setup, a stimulus isolator was used to pass constant direct current from anode to cathode via the mouse brain. Silver wire was used for both the anode and cathode. The anode and cathode sites were placed to ensure appropriate electrical stimulation of S1. The anode site consisted of an area of exposed skull located 2 mm posterior to the PSL-contralateral S1 (PSL injury at the right paw, anode placed near the left S1). During tDCS, the anode site was covered with conductive gel with the anode placed into this. The cathode site consisted of the neck region, with the cathode permanently sutured in place under the skin.

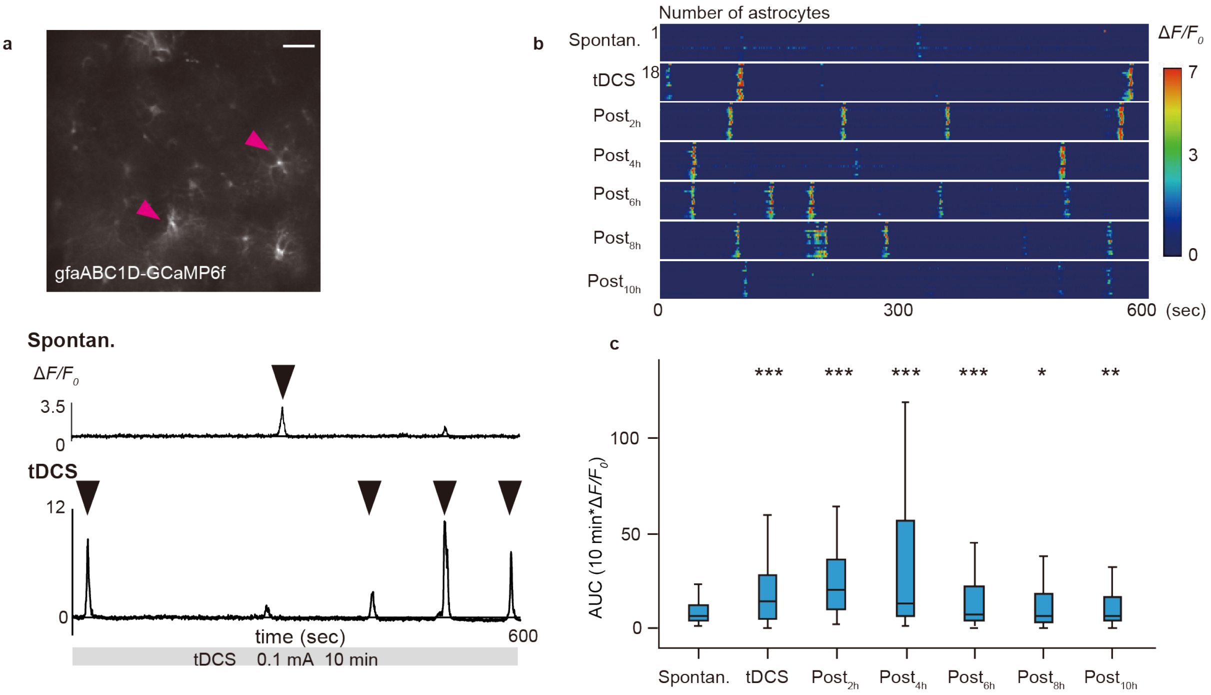

Supplementary Fig. 2. Astrocytic activation by tDCS

a. Upper panel: Representative image of S1 astrocytes expressing GCaMP6f. Imaging was conducted in awake mice through a cranial window using 2-photon microscopy. Two astrocytes from which Ca^{2+} events were observed are indicated by arrow heads. Scale bar = 20 μm. Lower panel: Sample Ca^{2+} fluorescence levels for a representative astrocyte illustrating Ca^{2+} transients (black arrow heads) under control conditions (upper trace, Spontan.) and during transcranial direct current stimulation (lower trace, tDCS of 0.1 mA for 10 minutes). This single tDCS session protocol was performed in 4 mice, with all showing reproducibility.

b and **c** A single session of tDCS induces prolonged increases in astrocytic Ca^{2+} activity. **b** An example raster plot of Ca^{2+} fluorescence over 10 minutes in 18 individual astrocytes from one mouse before (Spontan.), during (tDCS), and at different times after tDCS (Post 2 hrs – Post 10 hrs as indicated). **c** The sum of Ca^{2+} responses over 10 minutes (AUC) was significantly increased for up to 10 hours after tDCS (n = 132 astrocytes from 4 mice). Box and whisker plots indicate the population medians (horizontal lines), first and third quartiles (boxes), and maxima and minima (whiskers). Mean ± standard error (SEM) are as follows: pre: 9.3 ± 0.63, tDCS: 18.8 ± 1.5, post 2hr: 24.6 ± 1.5, post 4hr: 37.6 ± 4.0, post 6hr: 13.2 ± 1.1, post 8hr: 11.0 ± 0.85, post 10hr: 11.9 ± 1.1. Comparisons between Spontaneous Ca^{2+} activity (Spontan.) and all subsequent observation time points were tested using a one-way repeated measures ANOVA: p = 4.9E-86; followed by Bonferroni post-hoc tests, vs tDCS (p = 1.6E-15), vs 2 h (p = 1.4E-28), vs 4 h (p = 9.6E-29), vs 6 h

(p = 1.1E-07), vs 8 h (p = 1.6E-2), vs 10 h (p = 3.0E-3). Asterisks indicate the level of significance as follows: *$p < 0.05$, **$p < 0.01$, *** $p < 0.001$. Source data are provided as a Source Data file.

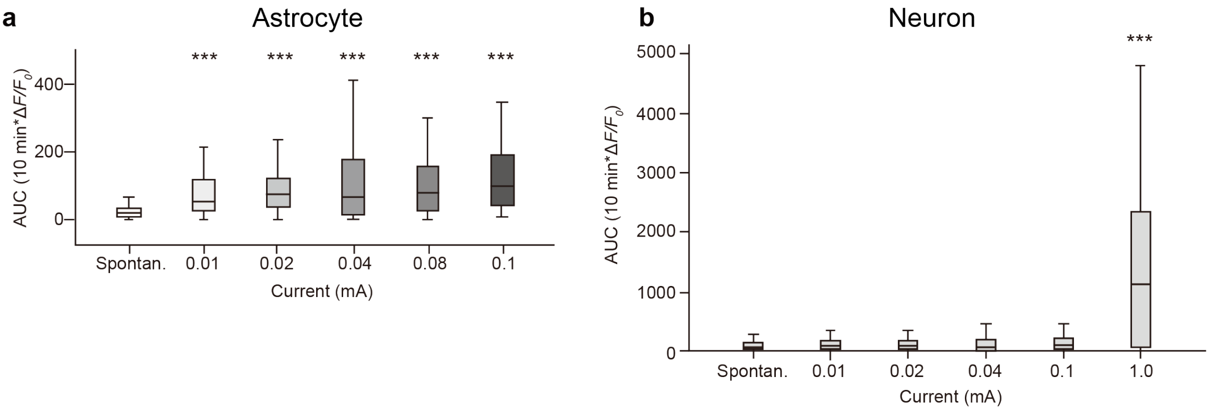

Supplementary Fig. 3. Astrocytic and neuronal Ca^{2+} responses at different tDCS current intensities.

a. Astrocytic Ca^{2+} activity ($n = 66$ astrocytes from 3 mice) was recorded in awake mice during tDCS. Different constant amplitudes, ranging from 0.01 mA to 0.1 mA, of the applied tDCS current were trialled in 10-minute sessions. Ca^{2+} activity for individual astrocytes was quantified by taking the integral of the normalized Ca^{2+} signal recorded over the entire 10-minute tDCS session. Astrocytic Ca^{2+} activity under all trialled tDCS current intensities was significantly increased as compared to astrocytic Ca^{2+} activity observed in the absence of tDCS (Spontan); one-way repeated measures ANOVA, interaction effect, $F(2.9, 90.8) = 29.6$, $p = 3.81E\text{-}13$; with Bonferroni post-hoc testing, vs 0.01 mA ($p = 1.5E\text{-}9$), vs 0.02 mA ($p = 2.1E\text{-}8$), vs 0.04 mA ($p = 3.1E\text{-}11$), vs 0.08 mA ($p = 2.1E\text{-}16$), vs 0.1 mA ($p = 8.7E\text{-}11$), *** $p < 0.001$. Based on this data, tDCS using a constant current of 0.1 mA for 10 minutes was used to induce elevated astrocytic Ca^{2+} activity in all subsequent experiments. Box and whisker plots indicate the population medians (horizontal lines), first and third quartiles (boxes), and maxima and minima (whiskers).. Source data are provided as a Source Data file. AUC, area under the curve.

b. Ca^{2+} activity ($n = 180$ neurons from 4 mice) was recorded from S1 cortical neuron somas in awake mice during tDCS. Different constant amplitudes, ranging from 0.01 mA to 1.0 mA, of the applied tDCS current were trialled in 10-minute sessions. Somatic Ca^{2+} activity for individual neurons was quantified by taking the integral of the normalized Ca^{2+} signal recorded over the entire 10-minute tDCS session. As compared to neuronal Ca^{2+} activity in the absence of tDCS (Spontan.), neuronal Ca^{2+} activity during tDCS was not elevated at current intensities less than 1.0 mA; one-way repeated measures ANOVA, interaction effect, $F(1.3, 2.4E+2) = 1.7E+2$, $p = 1.6E\text{-}35$; with Bonferroni post-hoc testing, vs 0.01 mA ($p = 1.0$), vs 0.02 mA ($p = 1.0$), vs 0.04 mA ($p = 1.0$), vs 0.1 mA ($p = 0.21$), vs 1.0 mA ($p = 1.6E\text{-}29$), *** $p < 0.001$. These experiments confirmed the astrocyte specificity of 0.1 mA current intensity tDCS. Box and whisker plots indicate the

population medians (horizontal lines), first and third quartiles (boxes), and maxima and minima (whiskers). Source data are provided as a Source Data file.

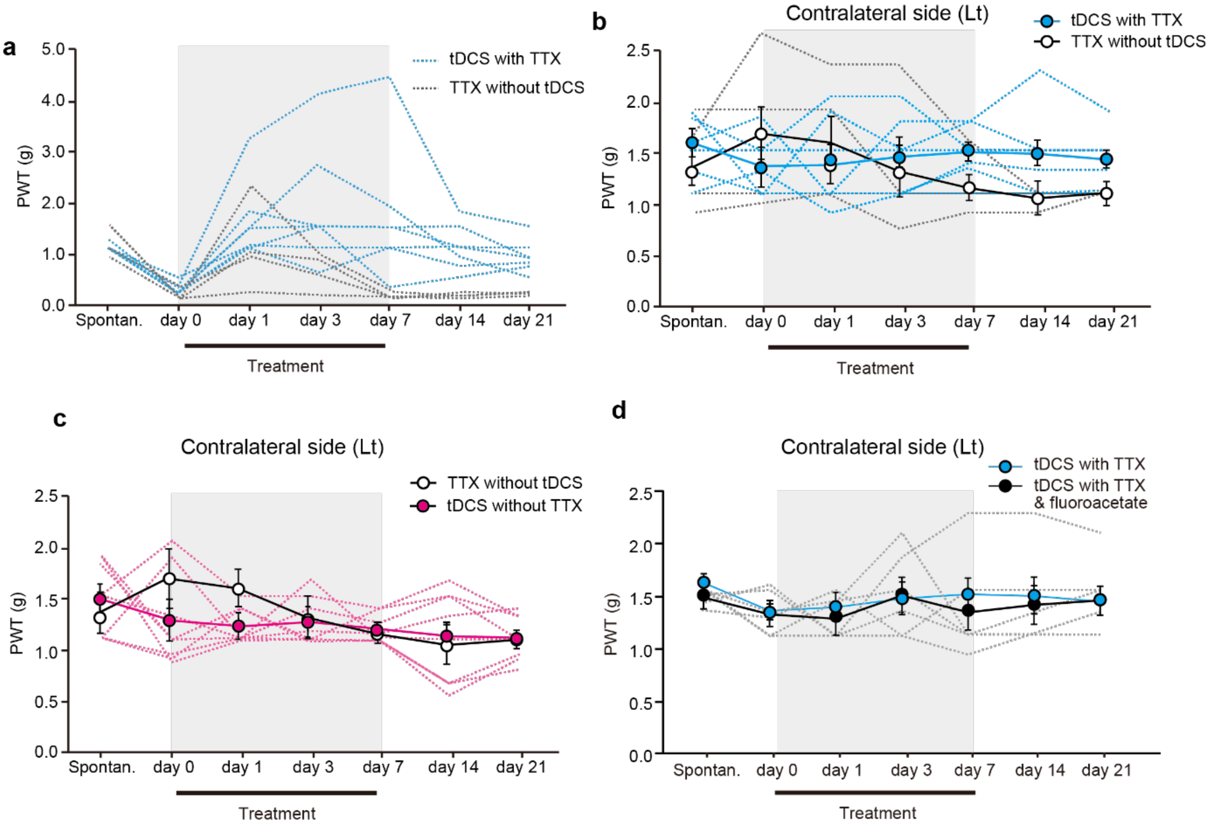

Supplementary Fig. 4. Withdrawal thresholds to mechanical stimuli in individual mice undergoing tDCS and/or TTX therapy.

a. In the main text, Fig. 1b plots the mean withdrawal thresholds of the PSL-affected (right) paw of tDCS with TTX mice and TTX without tDCS mice, which showed that PSL-induced mechanical allodynia-like behaviour is only reversed in tDCS with TTX mice. Here, the withdrawal thresholds of the individual mice from these same two cohorts are plotted in blue (tDCS and TTX) and black (TTX without tDCS), respectively. The treatment period (TTX and/or tDCS administration) is indicated by the grey shading. Source data are provided as a Source Data file. tDCS, transcranial direct current stimulation; TTX, tetrodotoxin; PWT, paw withdrawal threshold.

b. Withdrawal thresholds of the non-injured paw (contralateral to PSL, left) of tDCS with TTX mice and TTX without tDCS mice are plotted in blue and black, respectively. Solid lines plot the cohort means and standard errors, dotted lines plot the individual data (tDCS with TTX, $n = 6$; TTX without tDCS, $n = 4$). There was no significant difference in the withdrawal thresholds between the two cohorts; a two-way repeated measures ANOVA, the interaction effect, $F(2.7, 24) = 2.5$, $p =$ 8.7E-2. Source data are provided as a Source Data file.

c. Withdrawal thresholds of the non-injured paw (contralateral to PSL, left) of tDCS without TTX mice and TTX without tDCS mice are plotted in magenta and black, respectively. Note that the TTX

without tDCS data is the same as in Supp. 3b above. Solid lines plot the cohort means and standard errors, dotted lines plot the individual data (tDCS without TTX, $n = 8$; TTX without tDCS, $n = 4$). There was no significant difference in the withdrawal thresholds between the two cohorts; a two-way repeated measures ANOVA, interaction effect, $F(6, 60) = 1.4$, $p = 0.25$. Source data are provided as a Source Data file.

d. Withdrawal thresholds of the uninjured paw (contralateral to PSL, left) in tDCS with TTX & fluoroacetate mice and tDCS with TTX mice plotted in black and blue, respectively. Note that the tDCS with TTX data is the same as in Supp. 3b above. Solid lines plot the cohort means and standard errors, dotted lines plot the individual data (tDCS with TTX & fluoroacetate, $n = 6$; tDCS with TTX, $n = 7$). There was no significant difference in the withdrawal thresholds between the two cohorts; a two-way repeated measures ANOVA, the interaction effect, $F(3.4, 37) = 0.23$, $p = 0.97$. Source data are provided as a Source Data file.

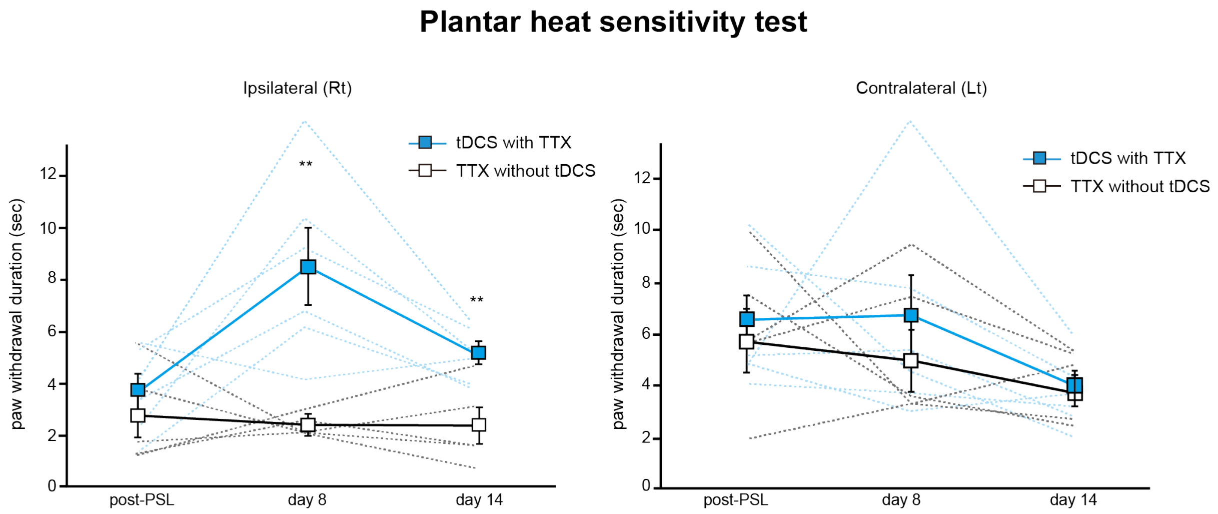

Supplementary Fig. 5. tDCS-TTX combination therapy also reverses thermal allodynia-like behaviour

In the plantar test, a non-nociceptive infrared heating beam is directed towards the mouse's paw. Thermal allodynia-like behaviour is indicated by a rapid paw withdrawal latency, similar to the von Frey test for mechanosensitive allodynia-like behaviour. The plantar test was conducted on tDCS with TTX and TTX without tDCS mice immediately post-PSL (prior to starting the 7 day treatment), and then 8 and 14 days after starting treatment. The left and right graphs show the withdrawal latencies of the injured (right, PSL-ipsilateral) and uninjured (left, PSL-contralateral) paws, respectively. The paw withdrawal latencies for tDCS with TTX and TTX without tDCS mice are plotted in blue and black, respectively. Solid lines plot the cohort means and standard errors, dotted lines plot the individual data (TTX with tDCS, $n = 6$; TTX without tDCS, $n = 5$). In the injured paw, the withdrawal latencies for tDCS with TTX mice were similar to TTX without tDCS mice immediately post-PSL but were significantly increased at both 8 and 14 days after starting treatment; a two-way repeated measures ANOVA, interaction effect, $F(2, 18) = 5.1$, $p = 1.7\text{E-}2$; with Bonferroni post hoc testing, post-PSL ($p = 0.40$), day 8 ($p = 4.0\text{E-}3$), day 14 ($p = 7.0\text{E-}3$), ** $p < 0.01$. In the uninjured paw, there was no significant difference in the paw withdrawal latencies between the two cohorts at any of the time points; a two way repeated measures ANOVA, interaction effect, $F(2, 18) = 0.24$, $p = 0.79$, main effect, $F(1, 9) = 0.77$, $p = 0.40$. Source data are provided as a Source Data file. tDCS, transcranial direct current stimulation; TTX.

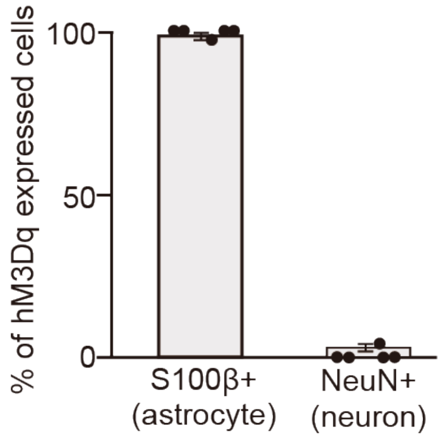

Supplementary Fig. 6. Selective expression of hM3Dq in S1 astrocytes

Immunohistochemistry showing selective expression of gfaABC1D-hM3Dq-mCherry in S1 astrocytes, as identified by S100β expression (see also Fig. 2a). Bar graphs plot the mean +/- SEM. Almost all S100β-positive cells (presumed astrocytes) also showed mCherry-expression (98.8±1.1%, n = 568 cells from 5 mice). In contrast, very few NeuN-positive cells (predominantly neurons) showed mCherry-expression (1.1±1.1%, n = 13 cells from 5 mice). Left figure scale bar = 500 μm (left figure). Right figure scale bar = 10 μm. Note that the representative immunohistochemistry image is included in the main text as within Figure 2a. Source data are provided as a Source Data file.

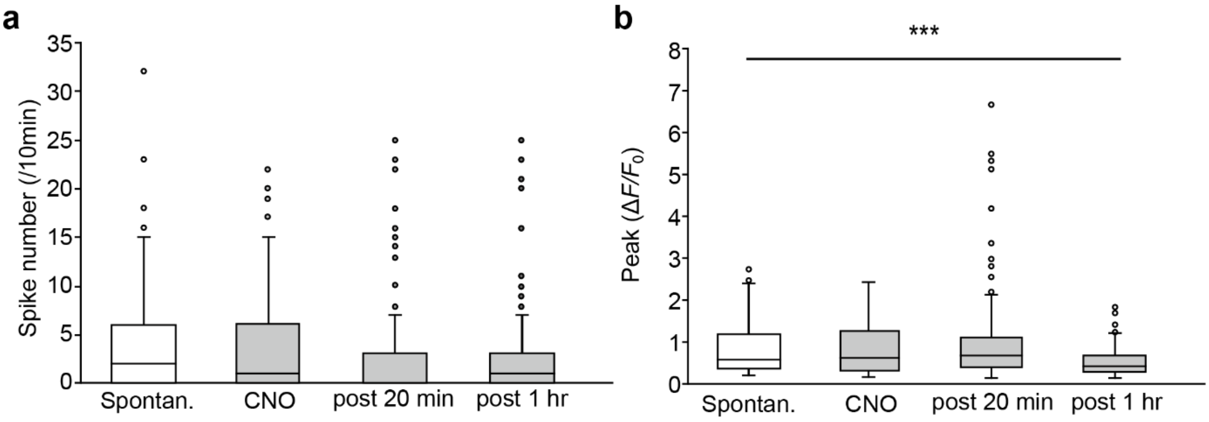

Supplementary Fig. 7. Neuronal activation was not induced by CNO injection.

Ca^{2+} imaging of S1 neurons was performed before and after CNO injection to confirm that neither S1 astrocyte activation by CNO nor CNO itself caused immediate off-target activation of S1 neurons. AAV-gfaABC1D-hM3Dq-mCherry and AAV-hSyn-GCaMP6f were injected into S1 to transfect resident astrocytes and neurons, respectively. Imaging was performed at a depth of 250-300 μm which corresponds to layer 3 of cortex.

a. The distribution of number of Ca^{2+} transients (spikes) recorded over a 10-minute period prior to (Spontan.), immediately after, 20 minutes after and 1 hour after CNO injection (n = 97 neurons from 3 mice). No changes in the number of Ca^{2+} transients were observed; a two way repeated measures ANOVA, interaction effect, $F(2.6, 251) = 23.0$, $p = 0.33$. Box and whisker plots indicate the population medians (horizontal lines), first and third quartiles (boxes), and maxima and minima (whiskers). Source data are provided as a Source Data file. CNO, clozapine N-oxide.

b. The distribution of peak amplitude of Ca^{2+} transients ($\Delta F/F_0$) recorded over a 10-minute period prior to (Spontan.), immediately after, 20 minutes after and 1 hour after CNO injection (n = 97 neurons from 3 mice). As compared to before CNO injection, peak $\Delta F/F_0$ was similar immediately after and 20 minutes after CNO injection but decreased 1 hour after CNO injection; a two way repeated measures ANOVA, interaction effect, $F(4,1813) = 33.9$, $p = 2.6E\text{-}27$; with Bonferroni post hoc testing, spontan. vs CNO, $p = 1.0$, spontan. vs post 20 min, $p = 0.21$, spontan. vs post 1hr, $^{***}p < 0.001$. Box and whisker plots indicate the population medians (horizontal lines), first and third quartiles (boxes), and maxima and minima (whiskers). Source data are provided as a Source Data file.

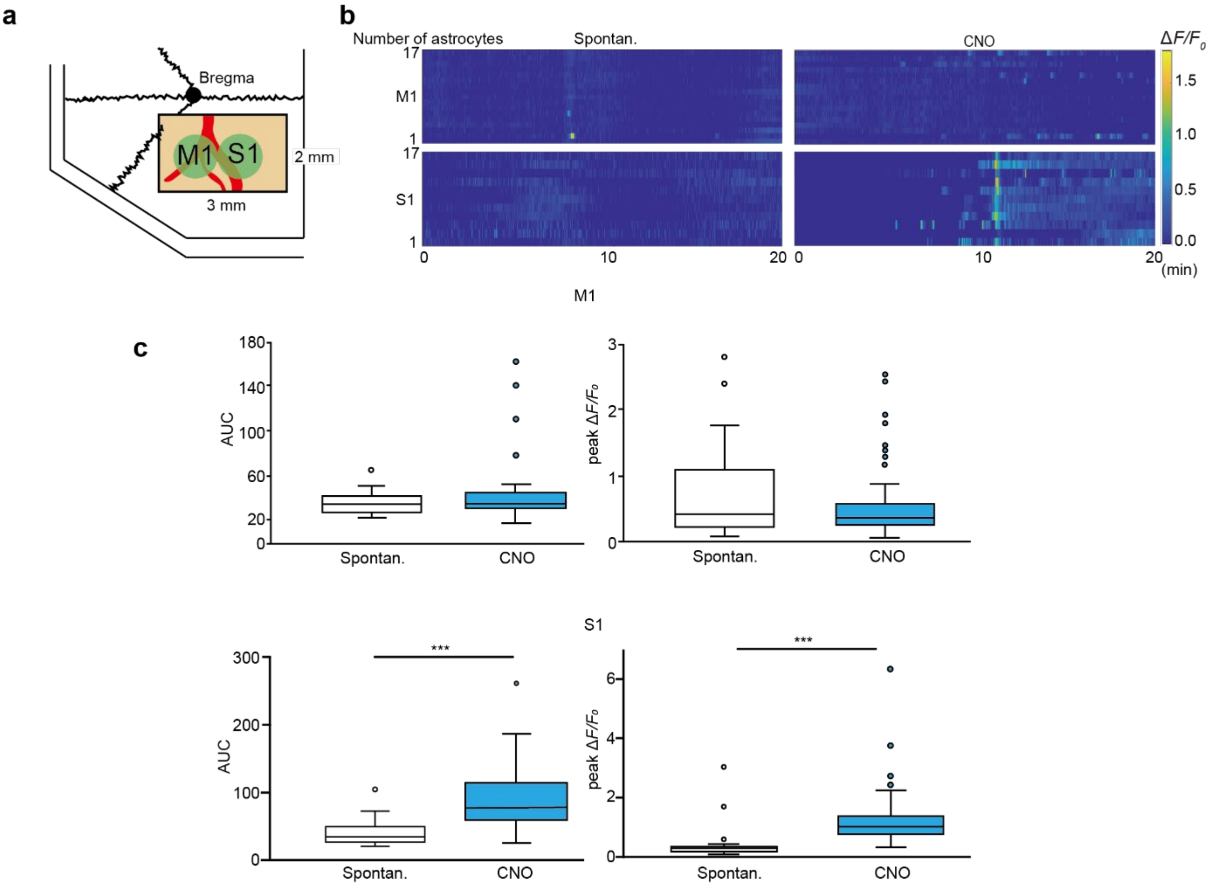

Supplementary Fig. 8. CNO injection increases the activitiy of S1 astrocytes which express hM3Dq but not of M1 astrocytes which do not express hM3Dq.

a. Schematic of M1 and S1 astrocyte Ca^{2+} imaging. To confirm that the therapeutic effect of CNO-TTX treatment is mediated by the exclusive activation of S1 astrocytes as opposed to global astrocyte activation, we performed Ca^{2+} imaging of M1 and S1 astrocytes following CNO injection. AAV-gfaABC1D-GCaMP6f was injected into both M1 and S1, while AAV-gfaABC1D-hM3D(Gq)-mCherry was injected into S1 only. Imaging of either M1 or S1 was performed before and after CNO injection. These M1 and S1 imaging sessions were performed in the same mice but were separated by at least 3 days to minimize any residual CNO effects.

b. Representative data of M1 and S1 astrocyte Ca^{2+} imaging from a single mouse. The relative change in Ca^{2+} activity for 17 individual astrocytes in either M1 (upper panels) or S1 (lower panels) over a 20 minute observation periods taken before (left panels) and after (right panels) a single CNO injection are plotted as heatmaps. A single injection of CNO induces an increase in S1 astrocytic Ca^{2+} activity, but does not change M1 astrocytic Ca^{2+} activity. CNO, clozapine N-oxide.

c. Distributions of signal integrals (left panels) and peak amplitudes (right panels) of the Ca^{2+} responses from M1 astrocytes (upper panels, n = 51 astrocytes from 3 mice) and S1 astrocytes

(lower panels, $n = 33$ astrocytes from 3 mice). For each individual astrocyte, these measures of Ca^{2+} transient activity were compared before and after a single injection of CNO. For M1 astrocytes, both the signal integrals (AUC) and peak amplitudes (peak $\Delta F/F_0$) of Ca^{2+} transients were the same before and after CNO; paired samples two-sided t-test for AUC, t(1.59) = 50, $p = 1.18E-1$; paired samples two-sided t-test for peak $\Delta F/F_0$, t(1.99) = 103, $p = 0.107$. For S1 astrocytic Ca^{2+} transients, both the mean integrals (AUC) and peak amplitueds ($\Delta F/F_0$)were elevated after CNO; paired samples two-sided t-test for AUC, t(5.51) = 32, $p = 4.0E-6$; paired samples two-sided t-test for peak $\Delta F/F_0$, t(1.99) = 81, p =1.18E-6, *** $p < 0.001$. Box and whisker plots indicate the population medians (horizontal lines), first and third quartiles (boxes), and maxima and minima (whiskers). Source data are provided as a Source Data file. AUC, area under the curve; Spontan., spontaneous; CNO, clozapine N-oxide

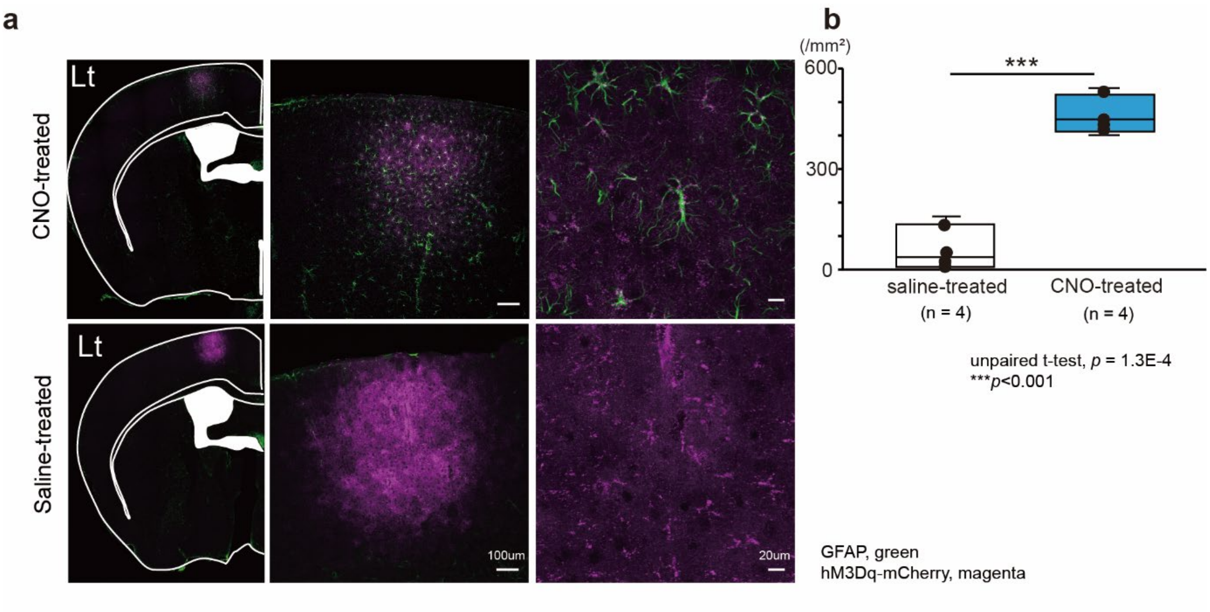

Supplementary Fig. 9. Repeated activation of S1 astrocytes induces strong GFAP expression. Repeated injections of CNO at 8-hourly intervals over 4 days induced intense GFAP staining in S1, indicating astrocyte activation. AAV-gfaABC1D-hM3Dq-mCherry was injected into the left S1 of all mice. Approximately 4 weeks later, mice had PSL performed on the right hind paw and were subsequently put on a CNO or saline injection regimen. Brains were perfused in preparation for GFAP immunohistochemistry 2 hours after the last CNO injection. CNO, clozapine N-oxide.

a. Immunohistochemistry of S1 cortex for GFAP (green) and mCherry-hM3Dq (magenta) in mice injected with CNO (upper panels) or saline (lower panels). Robust GFAP expression was only observed in CNO-treated mice whereas robust mCherry-hM3Dq expression was observed in both CNO-treated and saline-treated mice. Increasing magnifications of the left (AAV injected) S1 cortex are shown from left to right. The scale bars in the middle and right panels are 100 μm and 20 μm, respectively. For both cohorts, all sections ($n = 4$, both) showed reproducibility.

b. Box and whisker plots of GFAP-positive cell density in the left (AAV injected) cortex of hM3Dq + CNO + TTX ($n = 4$) and hM3Dq + saline + TTX ($n = 4$) mice. GFAP expression in hM3Dq + CNO + TTX mice was increased as compared to hM3Dq + saline + TTX mice; two-sided unpaired *t*-test, t(6) = 8.3, $p = 1.3E\text{-}4$, *** $p < 0.001$. Box and whisker plots indicate the population medians (horizontal lines), first and third quartiles (boxes), and maxima and minima (whiskers). Source data are provided as a Source Data file.

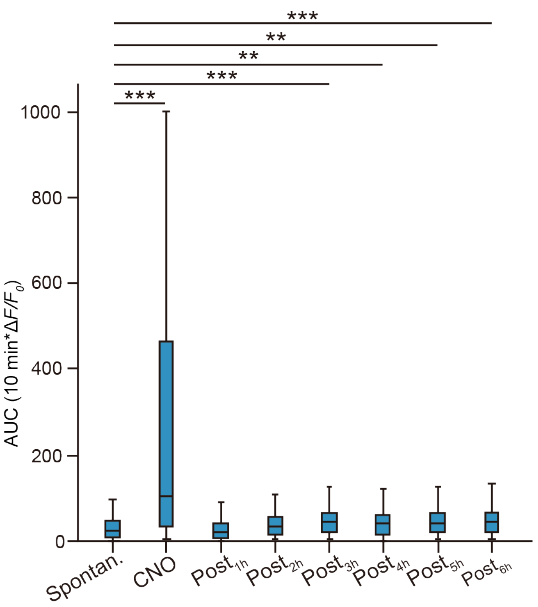

Supplementary Fig. 10. Prolonged increase in Ca^{2+} activity in astrocytes

A single injection of CNO (1.0 mg/kg) caused a prolonged increase in Ca^{2+} activity in astrocytes (*n* = 176 astrocytes from 6 mGFAP-cre mice). AAVs (CAG-flex-GCaMP6f and gfaABC1D-hM3Dq-mCherry) were injected into left S1 cortex. Ca^{2+} activity in astrocytes was observed for 10 minutes observation period (AUC) observed before (Spontan.), during (CNO), and at different times after (Post 1 hr to Post 6 hrs, as indicated), a single injection of CNO. Box and whisker plots of the summed Ca^{2+} activity over 10 minutes (AUC) indicate the population medians (horizontal lines), first and third quartiles (boxes), and maxima and minima (whiskers). Mean ± SEM are as follows: Spontan.: 33.2 ± 2.5, CNO: 478.8 ± 66.7, post 1hr: 29.7 ± 2.4, post 2hr: 41.4 ± 2.7, post 3hr: 49.4 ± 2.9, post 4 hr: 44.3 ± 2.6, post 5 hr: 44.9 ± 2.6, post 6 hr: 50.3 ± 2.9). Comparisons between control Ca^{2+} activity (Spontan.) and all subsequent observation time points were tested using a one-way repeated measures ANOVA: F (1.0, 1.8E+2) = 4.3E+01, p = 4.9E-10; followed by Bonferroni post-hoc tests, vs CNO (p = 8.0E-9), vs Post1h (p = 1.0), vs Post2h (p = 2.0E-1), vs Post3h (p = 2.0E-6), vs Post4h (p = 1.0E-2), vs Post5h (p = 3.0E-3), vs Post6h (p = 2.7E-7). ** $p < 0.01$, *** $p < 0.001$. Source data are provided as a Source Data file. AUC, area under the curve; Spontan.; spontaneous, CNO, clozapine N-oxide.

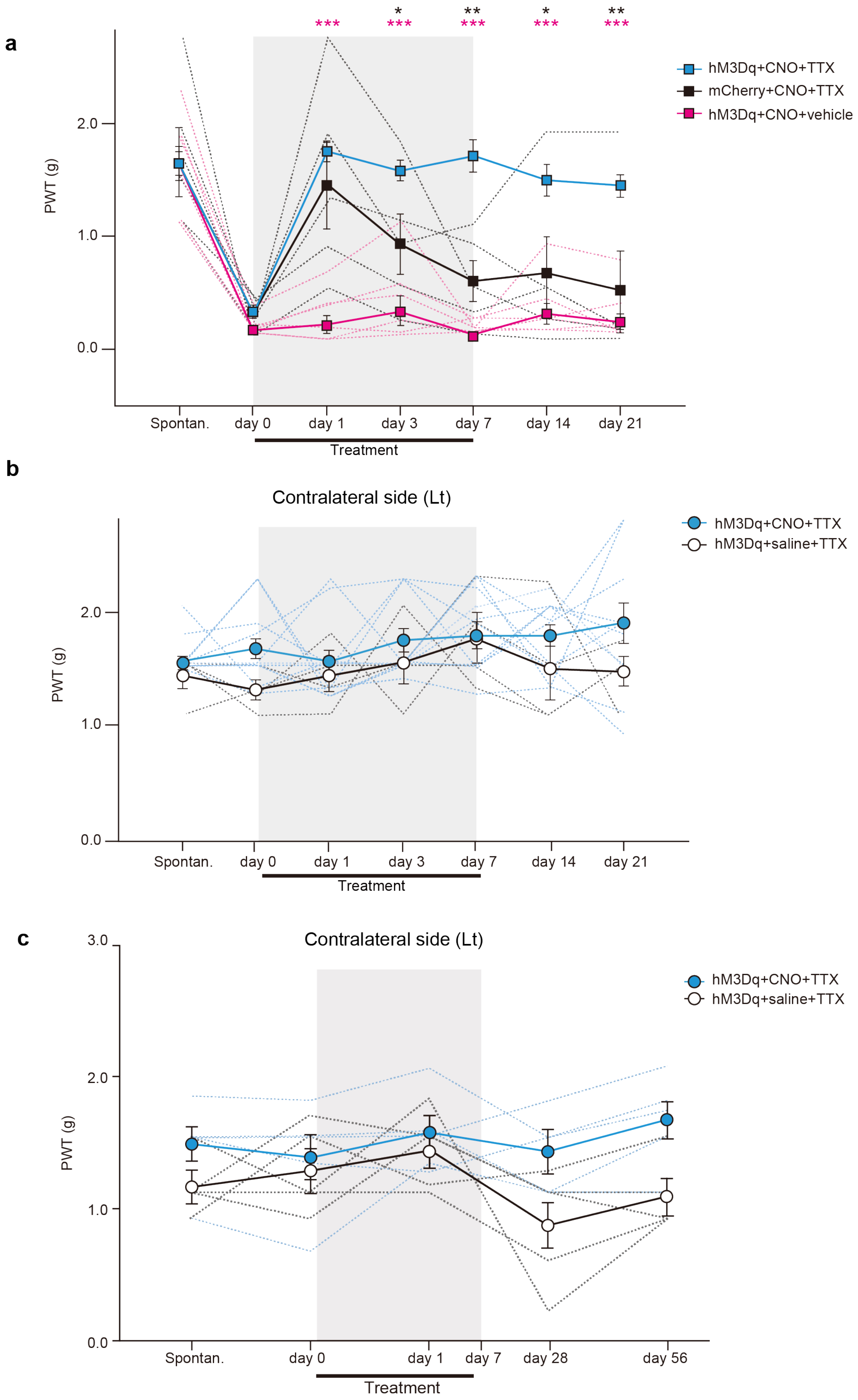

a
***
*
***
**
***
*
***
**
***
hM3Dq+CNO+TTX
mCherry+CNO+TTX
hM3Dq+CNO+vehicle
PWT (g)
2.0
1.0
0.0
Spontan.
day 0
day 1
day 3
day 7
day 14
day 21
Treatment
b
Contralateral side (Lt)
hM3Dq+CNO+TTX
hM3Dq+saline+TTX
PWT (g)
2.0
1.0
0.0
Spontan.
day 0
day 1
day 3
day 7
day 14
day 21
Treatment
c
Contralateral side (Lt)
hM3Dq+CNO+TTX
hM3Dq+saline+TTX
PWT (g)
3.0
2.0
1.0
0.0
Spontan.
day 0
day 1
day 7
day 28
day 56
Treatment

Supplementary Fig. 11. CNO (1.0 mg/kg) treatment only reverses allodynia-like behaviour in mice expressing hM3Dq in astrocytes and with concurrent TTX application.

a. In the main text, Fig. 2b plots the withdrawal thresholds of the PSL-affected (right) paw of hM3Dq + CNO + TTX mice showing the reversal of mechanical allodynia-like behaviour. This same data (hM3Dq + CNO + TTX, blue, $n = 13$) is plotted here along two additional control cohorts, (hM3Dq + CNO + vehicle, magenta, $n = 8$) and (mCherry + CNO + TTX, black, $n = 5$). The individual data of hM3Dq + CNO + TTX mice was also showed in Fig. 2b. Solid lines plot the cohort means and standard errors, dotted lines plot the individual data. The treatment period (TTX and/or CNO injection) is indicated by the grey shading. In hM3Dq + CNO + vehicle mice, CNO should still induce S1 astrocyte activation but there is no accompanying TTX treatment to block PSL-affected peripheral afferents. Compared to hM3Dq + CNO + TTX mice, the withdrawal thresholds of hM3Dq + CNO + vehicle remained hypersensitive at all times following PSL; a two-way repeated measures ANOVA, interaction effect, $F(7.3, 84.2) = 9.0$, $p = 2.1E\text{-}8$; with Bonferroni post hoc testing, Spontan. ($p = 1.0$), day 0 ($p = 3.9E\text{-}1$), day 1 ($p = 3.0E\text{-}7$), day 3 ($p = 1.0E\text{-}6$), day 7 ($p = 4.5E\text{-}8$), day 14 ($p = 5.4E\text{-}5$), and day 21 ($p = 7.0E\text{-}6$). In mCherry + CNO + TTX mice, CNO should no longer induce astrocyte activation but PSL-affected peripheral afferents were still temporarily blocked by TTX. Compared to hM3Dq + CNO + TTX mice, the withdrawal thresholds of mCherry + CNO + TTX mice also initially returned to pre-PSL levels (day 1) but then returned to the hypersensitive levels following loss of the TTX elution from the Elvax cuff (day 3 onwards); a two-way repeated measures ANOVA, interaction effect, $F(7.3, 84.2) = 9.0$, $p = 2.1E\text{-}8$; with Bonferroni post hoc testing, Spontan. ($p = 1.0$), day 0 ($p = 1.0$), day 1 ($p = 0.82$), day 3 ($p = 1.4E\text{-}2$), day 7 (p = 1.3E-4), day 14 ($p = 1.2E\text{-}2$), and day 21 ($p = 1.5E\text{-}3$). These results confirm mechanical allodynia-like behaviour is only reversed through simultaneous astrocyte activation with peripheral afferent blockade. The results also negate any potential non-astrocyte related effects of CNO or the AAV vector. *$p < 0.05$, ** $p < 0.01$, *** $p < 0.001$. Source data are provided as a Source Data file. CNO, clozapine N-oxide; TTX, tetrodotoxin; PWT, paw withdrawal threshold.

b. Mechanical withdrawal thresholds of the uninjured (contralateral to PSL, left) paw remain unchanged following PSL of the ipsilateral (right) paw and the subsequent CNO (1.0 mg/kg) treatment. Both mice cohorts were injected with the hM3Dq vector before undergoing PSL and subsequent TTX treatment, accompanied by either CNO (hM3Dq + CNO + TTX, blue, $n = 11$) or saline (hM3Dq + saline + TTX, black, $n = 4$) treatment. Solid lines plot the cohort means and standard errors, dotted lines plot the individual data. The treatment period (TTX and CNO/saline

administration) is indicated by the grey shading. There was no significant difference between the paw withdrawal thresholds following stimulation of the uninjured paw (left, PSL-contralateral) between the two cohorts before, during or after treatment; a two-way repeated measures ANOVA, interaction effect, $F(6, 78) = 0.484$, $p = 0.819$, main effect, $F(1,13) = 7.4$, $p = 1.8E-2$; with Bonferroni post hoc testing, Spontan. ($p = 1.3E-1$), day 0 ($p = 6.3E-2$), day 1 ($p = 5.2E-1$), day 3 ($p = 4.4E-1$), day 7 ($p = 9.1E-1$), day 14 ($p = 2.6E-1$), and day 21 ($p = 1.8E-1$). These results indicate that CNO itself has no effect on tactile and nociceptive perception processing independent of hM3Dq expression in S1 astrocytes. Source data are provided as a Source Data file.

c. In the long term, failure to treat mechanical allodynia-like behaviour in the PSL-affected paw leads to the development of mirror pain-like behaviour in the PSL-contralateral uninjured paw. The conditions and treatments of the two cohorts plotted here are the same as those in Supp. Fig. 11b above. Briefly, following PSL of the ipsilateral (right) paw, mice are treated with either 1.0 mg/kg CNO (hM3Dq + CNO + TTX, $n = 5$) or saline (hM3Dq + saline + TTX mice, $n = 5$) and the withdrawal thresholds of the PSL-contralateral uninjured (left) paw are measured over an extended 56-day period. As shown in the main text Fig. 2**c**, mechanical allodynia-like behaviour in the PSL-affected (right) paw is permanently cured in hM3Dq + CNO + TTX mice but permanently persists in hM3Dq + saline + TTX mice. Here, the withdrawal thresholds of the PSL-contralateral uninjured (left) paw for both cohorts are indicative of normal sensitivity to mechanical stimuli in the early period after PSL of the ipsilateral (right) paw. For hM3Dq + CNO + TTX mice, the withdrawal threshold remains constant throughout the extended observation period which suggests that CNO treatment produces no adverse effects on mechanosensation. For hM3Dq + saline + TTX mice, the withdrawal threshold trends towards hypersensitivity from day 28 onwards which suggests the development of pain-like behaviour; a two-way repeated measures ANOVA, interaction effect, $F(4, 32) = 1.52$, $p = 0.241$, main effect $F(4, 32) = 1.9$, $p = 0.13$. Source data are provided as a Source Data file.

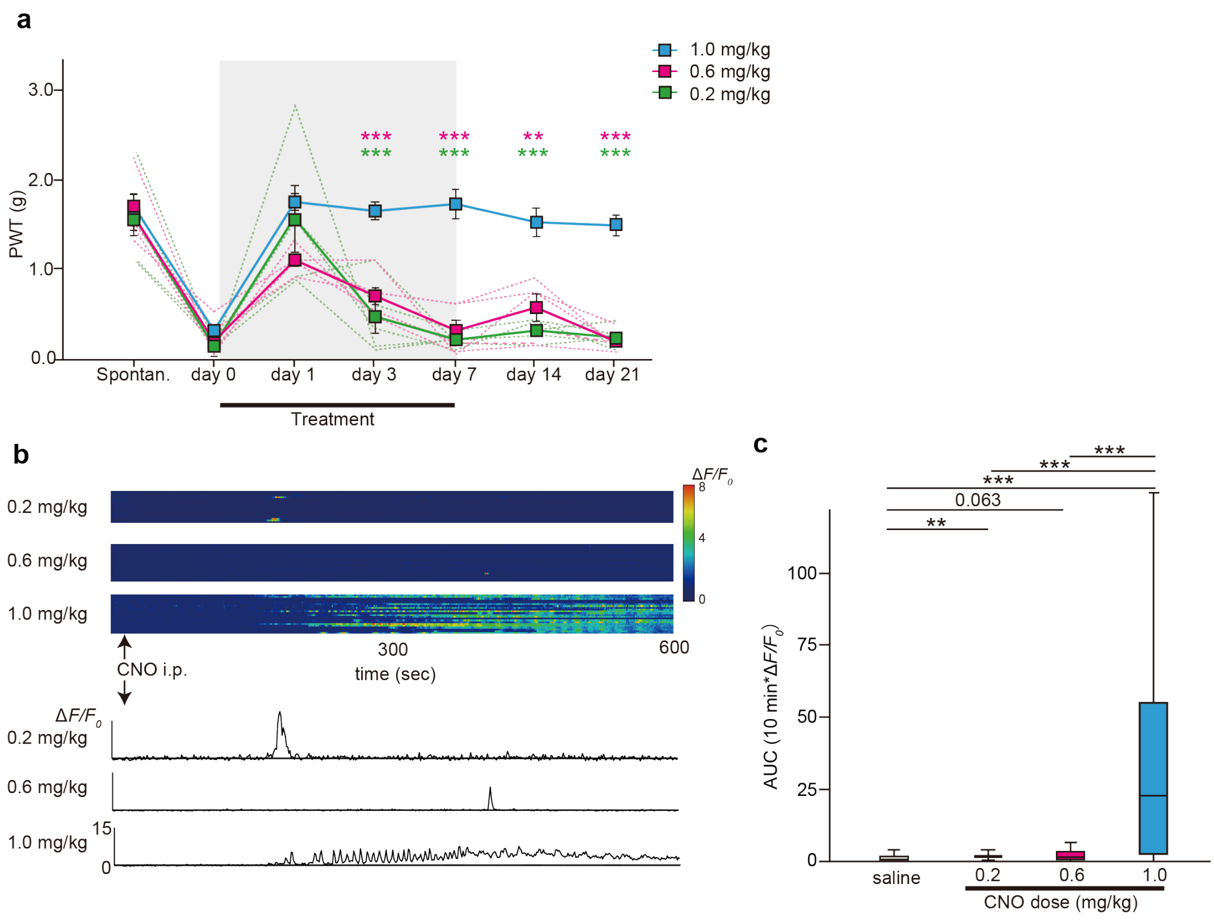

Supplementary Fig. 12. Optimizing the CNO dose to reverse allodynia-like behaviour. Using lower doses of CNO (0.2 or 0.6 mg/kg) in the treatment protocol failed to achieve long term reversal of allodynia-like behaviour. Consistently, the lower CNO doses elicited much smaller increases in astrocytic Ca^{2+} activity. ** $p < 0.01$, *** $p < 0.001$. CNO, clozapine N-oxide; PWT, paw withdrawal threshold; AUC, area under the curve.

a. Following PSL and subsequent TTX application to the injured (right) paw, mice expressing hM3Dq in astrocytes in the PSL-contralateral (left) S1 were injected with one of three doses of CNO, 1.0 mg/kg (blue, n = 12), 0.6 mg/kg (pink, n = 5) or 0.2 mg/kg (green, n = 5). The mean and standard error of the PSL-affected (right) paw withdrawal thresholds measured before and for 21 days after PSL are plotted in the solid lines, with individual mouse data in dotted lines. The treatment period is indicated by grey shading. Note that the data for 1.0 mg/kg CNO are the same as shown in Fig. 2b of the main text, replotted here for comparison. Compared to 1.0 mg/kg CNO-treated mice, the withdrawal thresholds of both 0.6 mg/kg and 0.2 mg/kg CNO-treated mice also initially returned to normal mechanosensation levels (day 1) but then returned to hypersensitive levels as TTX elution from the Elvax cuff subsided (day 3 onwards), so only 1.0 mg/kg produced sustained relief from allodynia-like behaviour; a two-way repeated measures ANOVA, interaction

effect, $F(8.4, 80.2) = 6.5$, $p = 1.0E-6$; with Bonferroni post hoc testing, Spontan. ($p = 1.0$ and $p = 1.0$), day 0 ($p = 0.24$ and $p = 0.78$), day 1 ($p = 1.0$ and $p = 5.9E-2$), day 3 ($p = 2.2E-4$ and $p = 9.0E-6$), day 7 ($p = 3.3E-5$ and $p = 1.5E-5$), day 14 ($p = 2.3E-3$ and $p = 2.0E-4$), and day 21 ($p = 6.4E-7$ and $p = 1.0E-6$); the first and second p-values per set refer to 1.0 mg/kg vs 0.6 mg/kg and 1.0 mg/kg vs 0.2 mg/kg, respectively. Source data are provided as a Source Data file.

b. The upper three heatmaps each correspond to individual representative mice expressing hM3Dq in S1 astrocytes, injected with either 0.2 mg/kg, 0.6 mg/kg or 1 mg/kg CNO. The heatmaps plot the relative changes in the Ca^{2+} activity of individual S1 astrocytes from each mouse over a 10-minute period following CNO injection. The lower three traces plot the $\Delta F/F_0$ of a single representative astrocyte from each of the three heatmaps over the same 10-minute observation period. Arrows indicate the time at which CNO was administered through intraperitoneal injection. During imaging of astrocytic Ca^{2+} transients, mice were anaesthetized with 1% isoflurane. All mice showed reproducibility.

c. Integrals of the individual Ca^{2+} transients in astrocytes (ROIs traced around the soma) were summed over the 10-minute observation period and were following either saline ($n = 88$ astrocytes from 3 mice) or CNO (0.2 mg/kg, $n = 52$ astrocytes from 3 mice; 0.6 mg/kg, $n = 48$ astrocytes from 3 mice; 1.0 mg/kg, $n = 76$ astrocytes from 3 mice) injection. 1.0 mg/kg CNO-treated mice exhibited significantly higher Ca^{2+} activity as compared with all other treatment cohorts with 0.6 mg/kg and 0.2 mg/kg CNO-treated mice also showing similar levels of activity to saline treated mice; Kruskal-Wallis H test, $p = 4.9E-23$; with Bonferroni post-hoc testing, saline vs 0.2 mg/kg ($p = 5.4-4$), saline vs 0.6 mg/kg (p =6.3E-2), saline vs 1.0 mg/kg ($p < 1.0E-25$), 0.2 mg/kg vs 1.0 mg/kg ($p = 2.0E-6$), and 0.6 mg/kg vs 1.0 mg/kg ($p = 3.9E-9$); **$p < 0.01$. ***$p < 0.0001$. Box and whisker plots indicate the population medians (horizontal lines), first and third quartiles (boxes), and maxima and minima (whiskers). Source data are provided as a Source Data file.

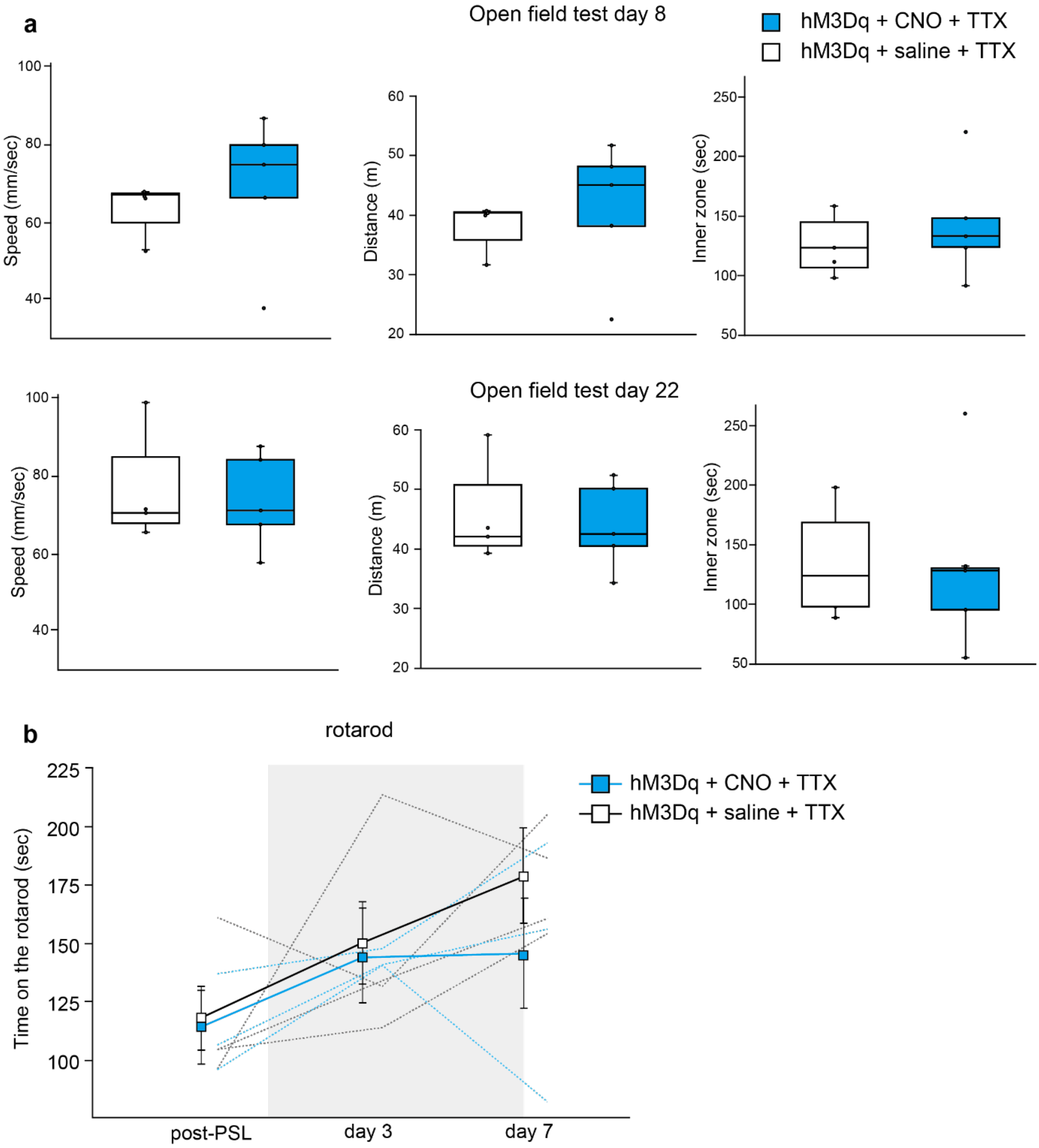

Supplementary Fig. 13. CNO-TTX therapy did not affect locomotive or anxiety-related behaviours.

a. Locomotion and anxiety behaviours were examined using the open field assay, where mice were placed in an uncovered 40 x 40 cm walled square arena and observed for 10 minutes. Locomotor behaviours quantified the average speed of movement (left panel) and the total distance travelled (centre panel) during the observation period, while anxiety was quantified by the total time spent within the centre of the open field arena (inner zone; right panel). PSL mice expressing hM3Dq in astrocytes in the contralateral S1 were tested one day (day 8; upper rows) and two weeks (day 22; lower rows) after the seven-day TTX and CNO (blue, $n = 5$) or TTX and saline (black, $n = 4$)

therapy. Day zero refers to 2 weeks after PSL, as in the protocol in the main text (see Fig. 2a). There were no significant differences in these parameters between the two cohorts; unpaired two-tailed *t*-test, speed (p = 1.0, day 8 and 22), distance (p = 1.0, day 8 and 22), time in inner zone (p = 1.0, day 8 and 22). Box and whisker plots indicate the population medians (horizontal lines), first and third quartiles (boxes), and maxima and minima (whiskers), with single points representing individual mice. Source data are provided as a Source Data file. CNO, clozapine N-oxide; TTX, tetrodotoxin. **b.** Motor ability was also assessed using the rotarod assay, where the rotation speed of a balancing beam was increased in a stepwise manner over 5 minutes, with the time taken for mice to fall off the beam quantified. PSL mice expressing hM3Dq in astrocytes in the contralateral S1 were subjected to the rotarod assay before therapy (day 0; post-PSL), three days after starting the CNO-TTX (blue, n = 3) or saline-TTX (black, n = 4) therapy (day 3), or after finishing therapy (day 7). Graph shows mean +/- SEM. The treatment period is indicated by grey shading. There was no significant difference between the two cohorts in the time taken to fall off the rotarod on either test day; a two-way repeated measures ANOVA, interaction effect: $F(2, 10) = 0.51$, $p = 0.617$, main effect: $F(1, 5) = 0.59$, $p = 0.48$. Source data are provided as a Source Data file.

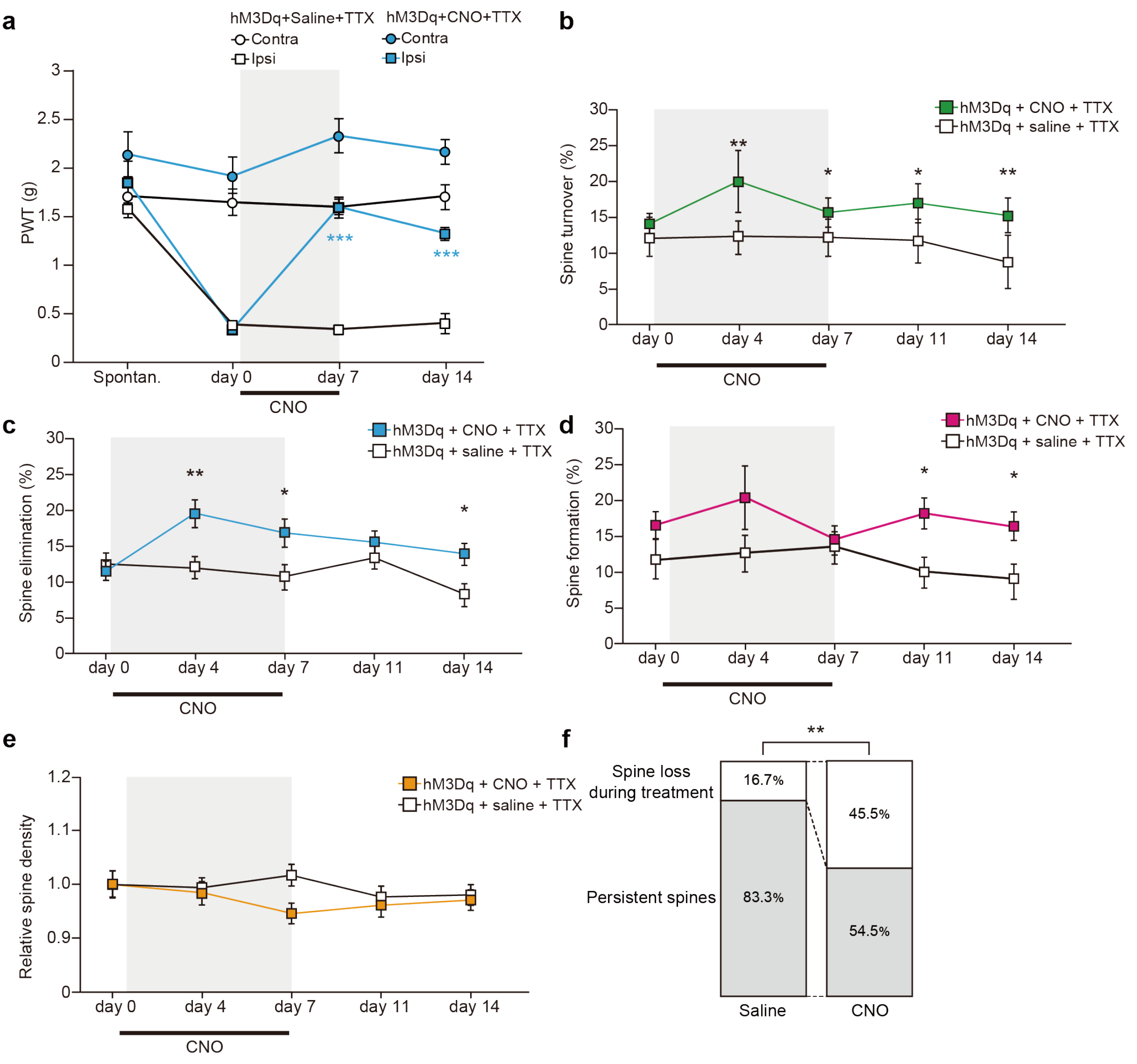

Supplementary Fig. 14. Expanded analysis of the CNO induced changes on spine dynamics. Fig. 3b-h of the main text shows key spine parameters for tDCS and CNO treatment groups, this data supplements that Figure showing absolute value for the experiments chemogenetically activating astrocytes with CNO. The treatment period is indicated by grey shading. CNO, clozapine N-oxide; TTX, tetrodotoxin; PWT, paw withdrawal threshold.

a. Withdrawal thresholds for both the injured paw (PSL-ipsilateral, right) and the uninjured (PSL-contralateral; left) paw were examined throughout the long-term spine imaging period. For all mice, hM3Dq was expressed in astrocytes in the PSL-contralateral (left) S1 cortex and TTX was applied via an elvax cuff to the PSL-affected (right) sciatic nerve. hM3Dq + CNO + TTX mice were injected with CNO (blue, n = 5), hM3Dq + saline + TTX mice were injected with saline (black, n = 7), both CNO-TTX or saline-TTX treatments lasted for seven days (grey shading) and began after confirming PSL had induced robust mechanical allodynia-like behaviour (day 0) on the injured

(right) paw. For both mice cohorts, TTX initially restored withdrawal thresholds to pre-PSL values, but this was only sustained for the hM3Dq + CNO + TTX mice; a two-way repeated measures ANOVA, F(3,30) = 8.8, p = 2.5E-4; with Bonferroni post hoc testing, Spontan. (p = 0.50), day 0 (p = 0.65), day 7 (p = 2.7E-5), and day 14 (p = 1.1E-4). The withdrawal thresholds for the uninjured (left) paw) never displayed mechanical allodynia-like behaviour in either group, as expected; a two-way repeated measures ANOVA, F(3,30) = 0.83, p = 0.49. ***p < 0.001. Graph shows mean +/- SEM. Source data are provided as a Source Data file.

b. to **d.** In the main text, Fig. 3 plots the spine turnover, elimination and formation rates as normalized to day 0 values. However, there is some variation amongst the CNO-treated mice (hM3Dq + CNO + TTX, n = 19 dendrites from 5 mice) and the saline-treated mice (hM3Dq + saline + TTX, n = 16 dendrites from 7 mice) and hence the absolute values of these measures are re-plotted here, and the two cohorts are compared at the different imaging days, *p < 0.05, **p < 0.01. The day 0 imaging was performed before the start of CNO-TTX or saline-TTX treatment, i.e. before the first CNO/saline injection (8-hourly injections over 7 days, grey shading). Graph shows mean +/- SEM.

b. Spine turnover rate was calculated as the sum of spines that were both formed and eliminated between two successive imaging sessions, divided by the doubled total number of spines counted in the prior imaging session. Spine turnover rates for hM3Dq + CNO + TTX mice (green) and hM3Dq + saline + TTX mice (white) were compared on each observation day; a two-way repeated measures ANOVA, interaction effect: F(3.1, 101) = 1.5, p = 0.22, main effect of treatment cohort: F(1, 33) = 19.3, p = 1.1E-4, with Bonferroni post hoc testing, day 0 (p = 0.28), day 4 (p = 3.5E-3), day 7 (p = 4.5E-3), day 11 (p = 1.0E-2), and day 14 (p = 4.0E-3). Graph shows mean +/- SEM. Source data are provided as a Source Data file.

c. Spine elimination rate was calculated as the number of spines that were lost between two successive imaging sessions divided by the total number of spines counted in the prior imaging session. Spine elimination rates for hM3Dq + CNO + TTX mice (blue) and hM3Dq + saline + TTX mice (white) were compared on each observation day; a two-way repeated measures ANOVA, interaction effect: F(4, 132) = 1.8, p = 0.13, main effect of treatment cohort: F(1, 33) = 11.9, p = 1.5E-3, with Bonferroni post-hoc testing, day 0 (p = 6.5E-1), day 4 (p = 5.2E-3), day 7 (p = 3.8E-2), day 11 (p = 0.47), and day 14 (p = 1.9E-2). Graph shows mean +/- SEM. Source data are provided as a Source Data file.

d. Spine formation rate was calculated as the number of spines that were formed between two successive imaging sessions, divided by the total number of spines counted in the prior imaging session. Spine formation rates for hM3Dq + CNO + TTX mice (magenta) and hM3Dq + saline + TTX mice (white) were compared on each observation day; a two-way repeated measures ANOVA, interaction effect: $F(2.8, 91.5) = 0.67$, $p = 0.61$, main effect of treatment cohort: $F(1, 33) = 18.0$, $p = 1.7E\text{-}4$; with Bonferroni post hoc testing, day0 ($p = 0.10$), day4 ($p = 0.14$), day7 ($p = 0.71$), day11 ($p = 1.3E\text{-}2$), and day14 ($p = 3.1E\text{-}2$). Graph shows mean +/- SEM. Source data are provided as a Source Data file.

e. Normalized spine density in hM3Dq + CNO + TTX mice (yellow; $n = 19$ dendrites from 5 mice) and hM3Dq + saline + TTX mice (white; $n = 16$ dendrites from 7 mice). Spine density was calculated by dividing the number of counted spines by the dendrite length. These values were then normalized against the day 0 spine density values. There were no significant differences in the normalized spine densities between hM3Dq + CNO + TTX mice (yellow) and hM3Dq + saline + TTX mice (white); a two-way repeated measures ANOVA, interaction effect: $F(3.2, 105.1) = 0.65$, $p = 0.60$. Graph shows mean +/- SEM. Source data are provided as a Source Data file.

f. Spines formed in the week following PSL, but before starting treatment, in hM3Dq + CNO + TTX mice ($n = 55$ spines from 19 dendrites, 5 mice) and in hM3Dq + saline + TTX mice ($n = 42$ spines from 16 dendrites, 7 mice) were divided into 2 categories based on whether they were lost during treatment (Spine loss during treatment) or were still present at 7 days after the end of treatment, i.e., at day 14 (Persistent). The proportions of these 2 categories were significantly different between hM3Dq + CNO + TTX mice and hM3Dq + saline + TTX mice, $\chi^2(1) = 8.928$, $p = 2.8E\text{-}3$, $^{**}p < 0.01$. Graph shows mean +/- SEM. χ^2 test is two sided. Source data are provided as a Source Data file.

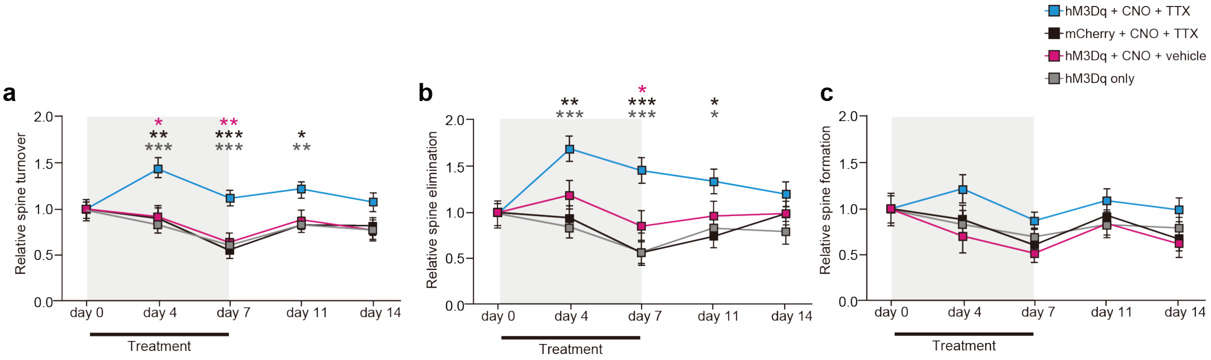

Supplementary Fig. 15. Spine dynamics in hM3Dq + CNO + TTX mice are heightened as compared to other control mice.

In the main text, Fig. 3 plots the long-term spine dynamics of hM3Dq + CNO + TTX mice and hM3Dq + saline + TTX mice in response to CNO-TTX and saline-TTX therapy, respectively. Here, the same spine dynamics data for hM3Dq + CNO + TTX mice (blue, $n = 19$ dendrites from 5 mice) is compared to three additional control cohorts which receive varying treatments following PSL. mCherry + CNO + TTX mice (black, $n = 14$ dendrites from 4 mice) control for CNO off-target effects of the CNO and TTX treatment. hM3Dq + CNO + vehicle mice (magenta, $n = 16$ dendrites from 3 mice) confirm the requirement for transient blockade of PSL-affected peripheral afferents by TTX to enable spine plasticity associated with therapy. hM3Dq only mice (grey, $n = 23$ dendrites from 4 mice) control for off-target effects caused by potential astrocyte activation arising from inflammation after AAV injection. *$p < 0.05$, **$p < 0.01$, ***$p < 0.001$. Note that the data for the hM3Dq+CNO+TTX mice are replotted from the same data in Fig. 3c-e. The treatment period is indicated by grey shading. Graph shows mean +/- SEM. CNO, clozapine N-oxide; TTX, tetrodotoxin.

a. Spine turnover rates of the three control cohorts were compared against hM3Dq + CNO + TTX mice on each observation day; a two-way repeated measures ANOVA, interaction effect: $F(11.8, 287.1) = 1.615$, $p = 8.8E-2$, main effect: $F(3, 73) = 10.7$, $p = 6.3E-6$; with Bonferroni post hoc testing, day 0 ($p = 1.0, 1.0, 1.0$), day 4 ($p = 3.2E-3, 1.1E-2, 4.0E-4$), day 7 ($p = 1.3E-4, 5.5E-3, 3.9E-4$), day 11 ($p = 2.0E-2, 1.3E-1, 1.6E-2$), and day 14 ($p = 3.8E-1, 3.2E-1, 1.7E-1$); the p-values in each set refer to hM3Dq + CNO + TTX vs mCherry + CNO + TTX, hM3Dq + CNO + TTX vs hM3Dq + CNO + vehicle and hM3Dq + CNO + TTX vs hM3Dq only, respectively. Source data are provided as a Source Data file.

b. Spine elimination rates of the three control cohorts were compared against hM3Dq + CNO + TTX mice on each observation day; a two-way repeated measures ANOVA, interaction effect: F(12,

292) = 1.83, p = 4.3E-3; with Bonferroni post hoc testing, day 0 (p = 1.0, 1.0, 1.0), day 4 (p = 1.2E-3, 1.3E-1, 1.5E-4), day 7 (p = 1.1E-4, 4.3E-2, 9.0E-5), day 11 (p = 1.0E-2, 4.1E-1, 4.5E-2), and day 14 (p = 1.0, 1.0, 1.5E-1); the p-values in each set refer to hM3Dq + CNO + TTX vs mCherry + CNO + TTX, hM3Dq + CNO + TTX vs hM3Dq + CNO + vehicle and hM3Dq + CNO + TTX vs hM3Dq only, respectively. Source data are provided as a Source Data file.

c. Spine formation rates of the three control cohorts were compared against hM3Dq + CNO + TTX mice on each observation day; a two-way repeated ANOVA, interaction effect: F(11.4, 277.4) = 0.42, p = 0.95, main effect: F(3,73) = 4.5, p = 5.6E-3; with Bonferroni post hoc testing, day 0 (p = 1.0, 1.0, 1.0), day 4 (p = 4.9E-1, 2.1E-1, 4.7E-1), day 7 (p = 2.0E-1, 6.2E-2, 8.3E-1), day 11 (p = 1.0, 1.0, 8.5E-1), and day 14 (p = 4.9E-1, 4.5E-1, 1.0); the p-values in each set refer to hM3Dq + CNO + TTX vs mCherry + CNO + TTX, hM3Dq + CNO + TTX vs hM3Dq + CNO + vehicle and hM3Dq + CNO + TTX vs hM3Dq only, respectively. Source data are provided as a Source Data file.

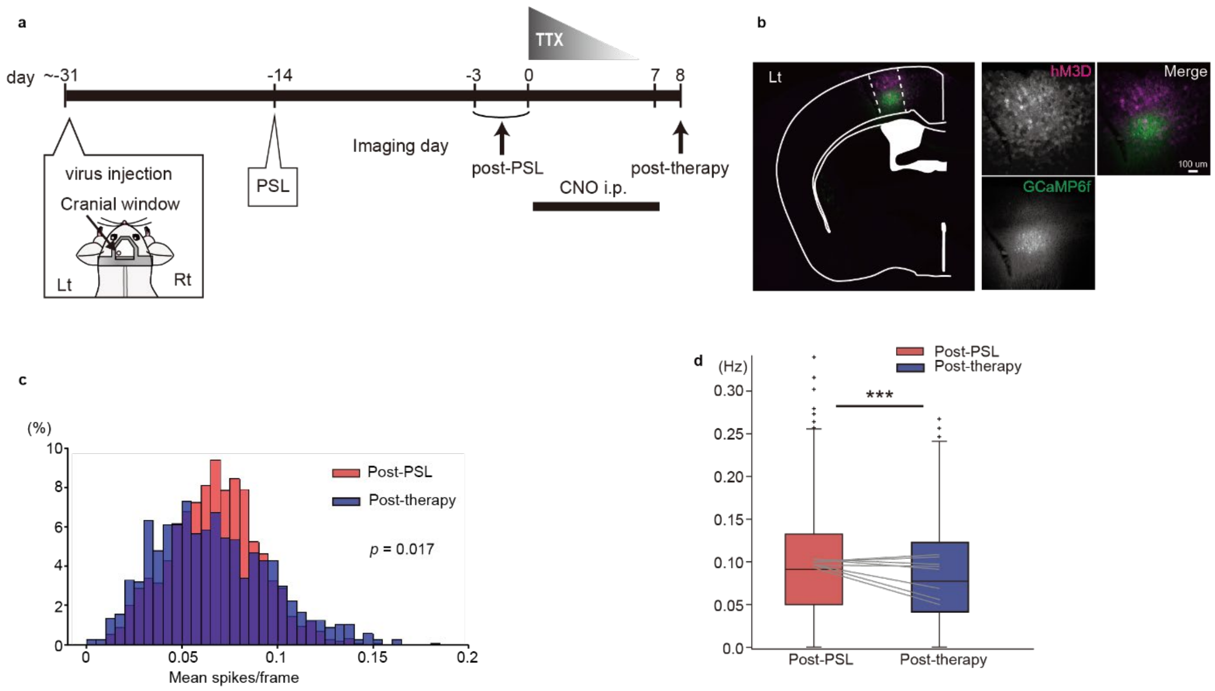

Supplementary Fig. 16. Influence of CNO-TTX therapy on neuronal Ca^{2+} activities in the PSL-contralateral S1 cortex

a. Ca^{2+} activity of L5 neurons in the PSL-contralateral (left) S1 cortex was observed using 2-photon microscopy in PSL-affected mice before and after CNO-TTX therapy. The schematic summarizes the experimental timeline with the two Ca^{2+} imaging sessions indicated by black arrows. At the first imaging session (Post-PSL), all mice exhibited mechanical allodynia-like behaviour. At the second imaging session (Post-therapy), all mice were cured of mechanical allodynia-like behaviour. PSL, partial sciatic nerve ligation; TTX, tetrodotoxin; CNO, clozapine N-oxide.

b. Sample immunohistochemistry image showing expression of hM3Dq-mCherry (magenta) and GCaMP6f (green) in the PSL-contralateral (left) S1 cortex. GCaMP6f was mainly expressed in layer 5 neurons whereas hM3Dq was mainly expressed in layer 3 astrocytes. All 8 sections showed same mCherry and GCaMP6f expression.

c. Distribution of neuronal activity before (Post-PSL, magenta, n = 1151 neurons from 8 mice) and after CNO-TTX therapy (Post-therapy, blue, n = 1031 neurons from 8 mice) as quantified by histograms of the mean frequency of Ca^{2+} transients in each neuron of S1. Mice were awake during the imaging session and only periods where mice were stationary have been used for analysis. Post-therapy, a significantly greater proportion of neurons showed a lower mean frequency of Ca^{2+} transients (mean spikes/frame), as compared to Post-PSL (before starting CNO-TTX therapy); two-sided Wilcoxon rank sum test, mean spikes/frame (p = 1.7E-2), positive correlation (p = 8.6E-4). Source data are provided as a Source Data file.

d. Neuronal activity in S1 during spontaneous locomotion decreased after CNO-TTX therapy. Box and whisker plots show the distribution of averaged frequency of neuronal Ca^{2+} transients for all individual imaged neurons before and after treatment while the connecting lines show the average Ca^{2+} transient frequencies from individual mice before and after treatment (post-PSL: n = 1151 neurons in 8 mice, post-therapy: n = 1031 neurons in 8 mice). Box and whisker plots indicate the population medians (horizontal lines), first and third quartiles (boxes), and maxima and minima (whiskers). Note how the mean frequency of Ca^{2+} transients was decreased in most (but not all) mice, with a significant decrease in the overall distributions. Therapy significantly reduced these parameters (two-sided Wilcoxon rank sum test, mean spikes (p = 2.5E-5), positive correlation (p =1.6E-12)). *** $p < 0.001$. Source data are provided as a Source Data file.

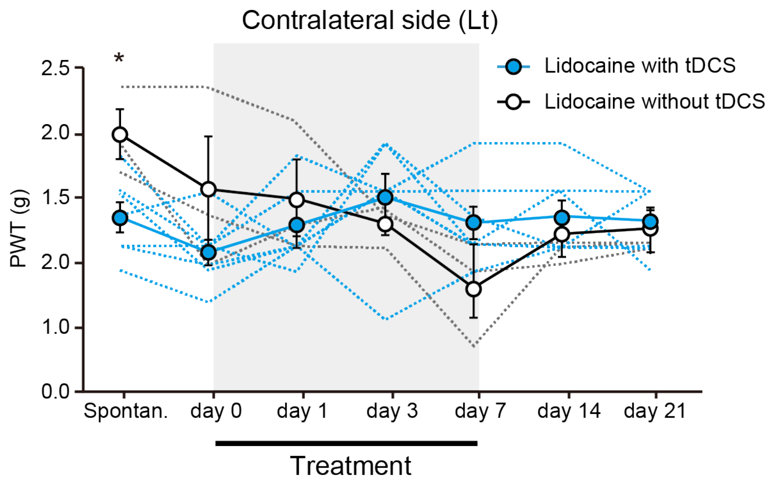

Supplementary Fig. 17. Mechanical thresholds of the PSL-contralateral uninjured paw are not affected by lidocaine with tDCS therapy.

In the main text, Fig. 5 demonstrates that lidocaine application to the PSL-affected (right) sciatic nerve in combination with tDCS stimulation of the PSL-contralateral (left) S1 cortex effectively cures mechanical allodynia-like behaviour. Here we show the effects of lidocaine on withdrawal thresholds of the uninjured (PSL-contralateral, left) paw. Withdrawal thresholds in mice treated with lidocaine and tDCS (blue, n =7), and mice treated with Lidocaine but without tDCS (white, n = 3), were not significantly different at any time after PSL, although there was a difference in initial thresholds between these cohorts, prior to any treatment.; a two-way repeated measures ANOVA, interaction effect, F(6, 48) = 5.21, p = 3.4E-4, with Bonferroni post hoc testing, Spontan. (p = 1.9E-2), day 0 (p = 0.13), day 1 (p = 0.38), day 3 (p = 0.47), day 7 (p = 7.3E-2), day 14 (p = 0.54), and day 21 (p = 0.74). *p < 0.05. Graph shows mean +/- SEM. Source data are provided as a Source Data file. tDCS, transcranial direct current stimulation; PWT, paw withdrawal threshold.
