# Supplementary material for: Controlled activation of cortical astrocytes modulates neuropathic pain-like behaviour
Source: Nat Commun. 2022 Jul 14;13:4100. doi: 10.1038/s41467-022-31773-8 (PMC9283422; doi:10.1038/s41467-022-31773-8)
Supplement: Supplementary file 2 — Reporting Summary [file 41467_2022_31773_MOESM2_ESM.pdf]

nature research

Corresponding author(s): Junichi Nabekura

Last updated by author(s): May 28, 2022

Reporting Summary

Nature Research wishes to improve the reproducibility of the work that we publish. This form provides structure for consistency and transparency in reporting. For further information on Nature Research policies, see our Editorial Policies and the Editorial Policy Checklist.

Statistics

For all statistical analyses, confirm that the following items are present in the figure legend, table legend, main text, or Methods section.

n/a	Confirmed	
☐	☒	The exact sample size (*n*) for each experimental group/condition, given as a discrete number and unit of measurement
☐	☒	A statement on whether measurements were taken from distinct samples or whether the same sample was measured repeatedly
☐	☒	The statistical test(s) used AND whether they are one- or two-sided<br>*Only common tests should be described solely by name; describe more complex techniques in the Methods section.*
☒	☐	A description of all covariates tested
☒	☐	A description of any assumptions or corrections, such as tests of normality and adjustment for multiple comparisons
☐	☒	A full description of the statistical parameters including central tendency (e.g. means) or other basic estimates (e.g. regression coefficient) AND variation (e.g. standard deviation) or associated estimates of uncertainty (e.g. confidence intervals)
☐	☒	For null hypothesis testing, the test statistic (e.g. *F*, *t*, *r*) with confidence intervals, effect sizes, degrees of freedom and *P* value noted<br>*Give P values as exact values whenever suitable.*
☒	☐	For Bayesian analysis, information on the choice of priors and Markov chain Monte Carlo settings
☒	☐	For hierarchical and complex designs, identification of the appropriate level for tests and full reporting of outcomes
☒	☐	Estimates of effect sizes (e.g. Cohen's *d*, Pearson's *r*), indicating how they were calculated

Our web collection on statistics for biologists contains articles on many of the points above.

Software and code

Policy information about availability of computer code

Data collection	Ca2+ imaging was performed using a Nikon A1R 2-photon microscope and accompanying NIS-Elements software (Nikon Instruments Inc.). Images for immunohistochemistry were acquired using a Nikon A1R confocal microscope and accompanying NIS-Elements software (Nikon Instruments Inc.).
Data analysis	AIVIA software (Version8.0 and 8.8, DRvision Technologies LLC, DC, USA) for spine analysis ; Matlab (R2014a, R2017b, Mathworks) for other analysis; ImageJ software environment (Version 1.51,1.52,1.53, NIH); SPSS (Version26,27,28)

For manuscripts utilizing custom algorithms or software that are central to the research but not yet described in published literature, software must be made available to editors and reviewers. We strongly encourage code deposition in a community repository (e.g. GitHub). See the Nature Research guidelines for submitting code & software for further information.

Data

Policy information about availability of data

All manuscripts must include a data availability statement. This statement should provide the following information, where applicable:

- Accession codes, unique identifiers, or web links for publicly available datasets
- A list of figures that have associated raw data
- A description of any restrictions on data availability

All data analysed during this study are included in this published article, its supplementary information files, and the Source Data. The raw data for two-phton imaging are available upon request.

Field-specific reporting

Please select the one below that is the best fit for your research. If you are not sure, read the appropriate sections before making your selection.

- [x] Life sciences
- [ ] Behavioural & social sciences
- [ ] Ecological, evolutionary & environmental sciences

For a reference copy of the document with all sections, see nature.com/documents/nr-reporting-summary-flat.pdf

Life sciences study design

All studies must disclose on these points even when the disclosure is negative.

Sample size	Sample size for each experiment is indicated in the figure legend for each experiment. The sample size was chosen based on previous experience for each experiment (Kim, et al., 2016) to ensure sufficiently high power for detecting statistically significant differences for each specific phenomena being studied. No statistical methods were used to predetermine sample size.
Data exclusions	On day 0 after PSL, mice with paw withdrawal thresholds over 0.6 g were excluded. This is because such mice are unlikely to develop allodynia which was the focus for the treatment that was developed by this study.
Replication	Fig1e shows that tDCS with TTX group is reproducible across 4 cohorts of mice (over 8 months). Fig1f shows that tDCS without TTX group is reproducible across 5 cohorts of mice.Fig1f shows that tDCS without TTX group is reproducible across 2 cohorts of mice. Fig2d and Supplementary Fig.11 shows that hM3Dq+CNO+TTX group is reproducible across 8 cohorts of BL6 and M-line mice by three different scientist. We checked twice as many mice as in other experiments and the results were the same. Fig2e shows that hM3Dq+CNO+TTX group is reproducible across 2 cohorts of mice. Fig4b shows that lidocaine with tDCS is reproducible across 3 cohorts of mice.
Randomization	All samples were allocated randomly by an experimenter who did not know the results of behavior.
Blinding	Investigators were blinded to group allocation during data analysis and data collection.

Reporting for specific materials, systems and methods

We require information from authors about some types of materials, experimental systems and methods used in many studies. Here, indicate whether each material, system or method listed is relevant to your study. If you are not sure if a list item applies to your research, read the appropriate section before selecting a response.

Materials & experimental systems

n/a	Involved in the study	
☐	☒	Antibodies
☐	☒	Eukaryotic cell lines
☒	☐	Palaeontology and archaeology
☐	☒	Animals and other organisms
☒	☐	Human research participants
☒	☐	Clinical data
☒	☐	Dual use research of concern

Methods

n/a	Involved in the study	
☒	☐	ChIP-seq
☒	☐	Flow cytometry
☒	☐	MRI-based neuroimaging

Antibodies

Antibodies used	rabbit anti-S100β (1:500; EP1576Y, ab52642, Abcam, Cambridge, UK,); mouse anti-NeuN (1:400; 1B7, ab104224, Abcam, Cambridge, UK); rabbit anti-GFAP (1:500; ab5804, Merck, Germany); goat anti-rabbit/anti-mouse secondary antibodies (1:300; Santa Cruz Biotechnology)
Validation	All of antibodies were validated by the manufacturers and have been cited by other authors. The related references are available on the relevant webpages of the provider companies. Abcam: their scientists will review the available literature to determine the best cell lines and tissues to use for validation. They then check the protein expression by IHC/ICC to see if it has the expected cellular localization. (https://www.abcam.com/primary-antibodies/how-we-validate-our-antibodies); Merck: They evaluated by immunohistochemistry on brain tissue, astrocytes and neurons. In addition, they have issued their own quality levels. anit-GFAP antibodies show highest quality level (https://www.merckmillipore.com/JP/ja/product/Anti-Glial-Fibrillary-Acidic-Protein-GFAP-Antibody,MM_NF-AB5804?ReferrerURL=https%3A%2F%2Fwww.google.com%2F); Santa Cruz:Their high quality, well characterized monoclonal secondary antibodies are available conjugated to either an enzyme, biotin or fluorophore for use in a variety of antibody-based applications including Western Blot, immunostaining and flow cytometry. Santa Cruz secondary antibodies are commonly affinity purified against immobilized whole IgG isotypes, including IgG1, IgG2a, IgG2b, IgG3 and IgG4 (https://www.scbt.com/ja/whats-new/immunocruz-antibody-conjugates).

Eukaryotic cell lines

Policy information about cell lines

Cell line source(s)	Human kidney embryo cell line 293 (HEK293)
Authentication	None of the cell line we used was authenticated.
Mycoplasma contamination	The cell line was not tested for mycoplasma contamination.
Commonly misidentified lines (See ICLAC register)	There is no misidentified line.

Animals and other organisms

Policy information about studies involving animals; ARRIVE guidelines recommended for reporting animal research

Laboratory animals	Male, 8-10 week old, C57BL/6 mice, M-line mice and mGFAP-Cre mice housed under a 12 hr light/dark cycle with free access to food and water at ~23 degrees celcius, ~30% humidity were used for all experiments.
Wild animals	This study did not involve wild animals.
Field-collected samples	This study did not involve samples collected from the field.
Ethics oversight	All animal experiments were approval by the Institutional Animal Care and Use Committee of the National Institutes for Natural Sciences(Approval numbers:.16A165, 17A137, 18A101, 19A091, 20A090, 21A049, 22A071).

Note that full information on the approval of the study protocol must also be provided in the manuscript.
